# Dynamics of T cell receptor distributions following acute thymic atrophy and resumption

**DOI:** 10.3934/mbe.2020002

**Published:** 2019-09-23

**Authors:** Stephanie M. Lewkiewicz, Yao-Li Chuang, Tom Chou

**Affiliations:** 1 Department of Mathematics, UCLA, Los Angeles, CA, 90095-1555, USA; 2 Department of Mathematics, CalState Northridge, Northridge, CA 91330, USA; 3 Department of Biomathematics, UCLA, Los Angeles, CA, 90095-1766, USA

**Keywords:** naive T cell diversity, clone abundance distribution, thymic output

## Abstract

Naive human T cells are produced and developed in the thymus, which atrophies abruptly and severely in response to physical or psychological stress. To understand how an instance of stress affects the size and “diversity” of the peripheral naive T cell pool, we derive a mean-field autonomous ODE model of T cell replenishment that allows us to track the clone abundance distribution (the mean number of different TCRs each represented by a specific number of cells). We identify equilibrium solutions that arise at different rates of T cell production, and derive analytic approximations to the dominant eigenvalues and eigenvectors of the mathematical model linearized about these equilibria. From the forms of the eigenvalues and eigenvectors, we estimate rates at which counts of clones of different sizes converge to and depart from equilibrium values—that is, how the number of clones of different sizes ”adjusts” to the changing rate of T cell production. Under most physiological realizations of our model, the dominant eigenvalue (representing the slowest dynamics of the clone abundance distribution) scales as a power law in the thymic output for low output levels, but saturates at higher T cell production rates. Our analysis provides a framework for quantitatively understanding how the clone abundance distribution evolves under small changes in the overall T cell production rate.

## 1. Introduction

The thymus, a small organ located above the heart in humans, is a crucial component of the primary lymphoid architecture, as the site of T cell development [1, 2]. The many different T cell subpopulations together guide and assist the action of other immune agents during infection [3], regulate the immune response [4], and retain memory of encountered pathogens [5]. As such, the thymus supplies the immune compartment with its most essential source of direction, support, and regulation. T cells are produced when lymphocyte progenitors derived from hematopoietic stem cells in the bone marrow migrate to the thymus and begin a process of role selection, maturation, and vetting, before being exported to the peripheral blood [6]. The most significant event during thymocyte development is the rearrangement of the *α* and *β* chains of the T cell receptor (TCR) [7] that occurs in the thymic cortex, which is populated with thymocyte progenitors. The particular rearrangement a T cell undergoes determines its antigen specificity; a naive T cell in the peripheral pool is activated when its TCR is bound by a cognate antigen, a pathogen-derived peptide fragment capable of stimulating that particular TCR [8]. The total number of distinct TCRs present across the full T cell pool is the “TCR diversity” [9], and this quantifies the breadth of the pool’s antigen responsiveness [10]. Thymocytes also undergo negative selection to eliminate cells that react too strongly to self antigens presented by resident macrophages and dendritic cells. The small number of T cells that survive this process are functionally competent and thus exported to the peripheral blood to participate in the immune mechanism.

The thymus is known to experience both chronic and acute forms of atrophy [11], resulting from both normal biological processes and the presence of disease or stress. The most universal form of thymic atrophy is age-related involution, the process by which productive thymic tissue is gradually replaced with nonproductive fat [12]. Involution begins at puberty and continues indefinitely, and the resulting decline in T cell production has been implicated as a likely source of immune dysfunction in the elderly [13–15]. Acute atrophy can occur under a plethora of conditions associated with a state of disease or stress [16–18], including viral, bacterial and fungal infection [19–21], malnutrition [22], cancer treatment [23], bone marrow transplant [24], psychological stress and pregnancy [25–27]. Each condition facilitates thymic atrophy in (at least) one of several ways, including reducing thymic cellularity [11], decreasing thymocyte proliferation and increasing apoptosis [28], instigating premature export of underdeveloped thymocytes [29], and inducing morphological changes to the thymic microenvironment [30]. Such disturbances may consequently alter the size and composition of the peripheral T cell pool. Decreased lymphocyte prevalence in the periphery during acute involution has been documented [31–34], and *Salmonella,* which infects the thymus itself, has been shown to disrupt positive and negative selection, producing a skewed TCR repertoire [35]. Radiation and chemotherapy drugs, such as temozolmide, used to treat cancer can also be highly lymphotoxic, producing a lymphopenic state referred to as “treatment-related lymphopenia” (TRL) [36–38]. Viral infections, particularly HIV, and autoimmune disorders can induce lymphopenia by increasing peripheral cellular death and redistributing cells to inappropriate tissues, in addition to affecting production in the thymus [39]. Congenital thymic aplasia, as seen in complete DiGeorge syndrome, results in a lymphopenic state at birth [40].

The activation of the hypothalamic-pituitary-adrenal axis by stress stimuli and subsequent release of glucocorticoids, which are known to induce apoptosis in double-positive thymocytes [41] and inhibit their differentiation [28], is also likely a major underlying catalyst of this acute involution [17, 42]. Evidence suggests that glucocorticoid release is actually necessary to affect thymic atrophy [18, 28]. Several other chemical agents have been observed to participate in thymic atrophy, notably sex hormones [17], which have been shown to weaken thymocyte proliferation [25] and induce apoptosis [43], and the IL-6 cytokine family, which is demonstrably thymosuppressive [16]. Despite this apparent sensitivity to stress, the thymus is highly plastic, and generally recovers in size and functionality after removal of the stressor [11]. Studies of the thymus during and after chemotherapy treatment in cancer patients indicate a return of thymic volume and productivity during recovery from treatment [23]. A recuperating thymus may even surpass its pre-treatment volume, in a phenomenon known as “thymic rebound” [44,45]. Such thymic recovery has also been seen after infection [46] and traumatic injury [47]. However, recovery is demonstrably age-dependent, with thymi of older patients reconstituting the naive T cell compartment more weakly than those of younger patients [23]. Although acute thymic atrophy has been observed extensively in humans, much has yet to be learned about it, and clear treatment protocol is lacking [11].

To this end, we present a mechanistic mathematical model to predict changes in the size and diversity of the peripheral naive T cell compartment in response to various immunologically diseased conditions. We study how this pool’s size and composition adjust to changes in the rate of thymic output. We compartmentalize the peripheral T cell pool by grouping clones–collections of T cells with the same TCR–according to their size. We then use a high-dimensional autonomous ODE system to follow the time evolution of the number of clones in each compartment. We assume that the size of the peripheral naive T cell pool is dictated by rates of thymic export of new T cells, along with homeostatic proliferation and death mechanisms. We assume a piecewise constant rate of thymic export, as the atrophy/recovery cycle is known to be a rapid process, and that the proliferative and death processes are subject to homeostatic regulation based on the total T cell pool size. We derive analytic approximations to the dominant eigenvalues and eigenvectors of the system linearized around its equilibria in both the presence and the absence of thymic activity. From this, we assess the rates of convergence of different T cell compartments to equilibria that result from a changing thymic export rate. We then compare the linearized and fully nonlinear models, and study several special cases. We also compute explicit representations of solutions to an infinite-dimensional extension of our model.

## 2. Mathematical model and analysis

We assume that the total naive T cell population *N*(*t*) in the immune compartment (the blood and lymphatic tissue) satisfies a general ODE of the form

(1) $$\frac{\text{d}N}{\text{d}t}=\gamma +p(N)N-\mu (N)N,$$

where *γ* ≥ 0 is the rate of naive T cell export from the thymus, and *p*(*N*), *μ*(*N*) ≥ 0 are regulated, *N*-dependent rates of proliferation and death of naive T cells in the peripheral bloodstream. To prevent unbounded growth, we take *p*(*N*) to be non-increasing and *μ*(*N*) to be non-decreasing as cell counts, *N*, increase. We assume that *p*(0) > *μ*(0), as the lymphopenic proliferation rate should be higher than the lymphopenic death rate [48–51]. At steady-state, when a healthy, homeostatic cell count N* is achieved, $p\left(N^{*}\right)-\mu \left(N^{*}\right)=-\frac{\gamma }{N^{*}}\leq 0$. Note that when *γ* = 0, simple decreasing functions *p*(*N*) (and/or increasing functions *μ*(*N*)) admit multiple–typically two–fixed points. The *N* = 0 fixed point is unstable, while the one at *N* > 0 is stable.

In order to compute the peripheral naive T cell diversity, we couple Eq 1 with a system of ODEs that describes the time evolution of the size-segregated subpopulations of the peripheral naive T cell pool. Let *c*_*k*_(*t*) denote the number of clones that are of size *k* at time *t* ≥ 0. As formally shown in Song & Chou [52], the *mean* clone count is $c_{k}\propto P\left(n_{i}=k,t\right),$ the marginalized probability that *any* single clone *i* has population *k*. The master equation for *P*(*n*_*i*_, *t*) is difficult to solve with regulation terms. Here, we provide a heuristic derivation of the equations obeyed by *c*_*k*_(*t*) by using a mean-field approximation that the *total* population *N* is uncorrelated with any *n*_*i*_, (although we know $N=\sum_{i=1}^{\infty }n_{i}$). Under this mean-field approximation, the evolution of *P*(*n*_*i*_ = *k*, *t*), and hence *c*_*k*_(*t*), obeys

(2) $$\frac{\text{d}c_{1}}{\text{d}t}=\frac{\gamma }{\Omega }\left[\Omega -\sum_{i=1}^{M}c_{i}-c_{1}\right]-p(N)c_{1}+\mu (N)\left[2c_{2}-c_{1}\right],$$

(3) $$\frac{\text{d}c_{k}}{\text{d}t}=\frac{\gamma }{\Omega }\left[c_{k-1}-c_{k}\right]+p(N)\left[(k-1)c_{k-1}-kc_{k}\right]+\mu (N)\left[(k+1)c_{k+1}-kc_{k}\right],$$

(4) $$\frac{\text{d}c_{M}}{\text{d}t}=\frac{\gamma }{\Omega }c_{M-1}+p(N)(M-1)c_{M-1}-\mu (N)Mc_{M},$$

where *k* = 2, 3,..., *M* − 1, *p*(*N*) and *μ*(*N*) are approximated by *p*(*N*(*t*)) and *μ*(*N*(*t*)) (where *N*(*t*) is the solution to Eq 1), and the index *M* in Eq 4 is the hypothetical maximum size a clone can achieve. We take *M* to be finite for mathematical tractability and in accordance with evidence of intraclonal competition that restricts clone sizes and preserves a balanced TCR diversity [53]. Each of the ODEs in Eqs 2–4 describes how *c*_*k*_ changes due to the effects of thymic export of new cells, and proliferation and death in the periphery. The constant Ω denotes the large total number of clonotypes that can potentially be assembled in and exported from the thymus. The basic model includes immigration, birth, and death of multiple species (*i.e.*, clones) and can be developed in a fully stochastic setting; however, in that case, only steady-state solutions are available [54].

In Eq 3, the term $\frac{\gamma }{\Omega }$ represents the rate at which cells of a given clonotype are exported to the periphery from the thymus, and thus $\frac{\gamma }{\Omega }c_{k}$ represents the export rate of cells of clonotypes already represented by size-*k* clones in the periphery. The addition of a new cell to a clone of *k* cells decreases by one the number of clones with *k* cells and increases by one the number of clones with *k* + 1 cells. Similarly, $\frac{\gamma }{\Omega }c_{k-1}$ represents the rate at which clones move from *c*_*k* − 1_ to *c*_*k*_ due to thymic export. We assume that cells immigrate, proliferate, or die one cell at a time, forcing clones to move only among adjacent compartments. Thus, the term $\frac{\gamma }{\Omega }\left[c_{k-1}-c_{k}\right]$ fully accounts for changes to *c*_*k*_ due to thymic export. The term *p*(*N*)*kc*_*k*_ denotes the rate at which cells in size-*k* clones proliferate, which in turn corresponds to the rate at which clones move from *c*_*k*_ to *c*_*k* + 1_ due to peripheral proliferation. Analogously, *p*(*N*)(*k* − 1)*c*_*k* − 1_ denotes the rate at which clones enter *c*_*k*_ from *c*_*k* − 1_ due to proliferation, so that *p*(*N*)[(*k* − 1)*c*_*k* − 1_ − *kc*_*k*_] accounts for changes to *c*_*k*_ due to proliferation. The death term in Eq 3, given by *μ*(*N*) [(*k* + 1)*c*_*k* + 1_ − *kc*_*k*_], is defined analogously. Eqs 2 and 4 represent “boundary conditions” for Eq 3. In Eq 2, the term $\left[\Omega -\sum_{i=1}^{M}c_{i}\right]$ gives the number of clonotypes unrepresented in the periphery, so that $\frac{\gamma }{\Omega }\left[\Omega -\sum_{i=1}^{M}c_{i}\right]$ provides the rate at which new clones enter the periphery from the thymus. Equation 2 also retains the terms from Eq 3 that account for loss of clones in *c*_1_ due to thymic export, proliferation, and death, and the addition of clones into *c*_1_ due to death of cells in *c*_2_. Finally, Eq 4 retains terms accounting for the introduction of clones into *c*_*M*_ via thymic export to and proliferation within clones in *c*_*M* − 1_, as well as loss of clones from *c*_*M*_ due to cellular death. This represents a “boundary condition” that prevents clones from surpassing size *M*.

Summing Eqs 2–4, we find that

(5) $$\frac{\text{d}\left(\sum_{k=1}^{M}kc_{k}\right)}{\text{d}t}=\gamma +p(N)\left(\sum_{k=1}^{M}kc_{k}\right)-\mu (N)\left(\sum_{k=1}^{M}kc_{k}\right)-\left(p(N)Mc_{M}+\frac{\gamma_{0}}{\Omega }c_{M}\right),$$

so that the ODE satisfied by *N*(*t*) (Eq 1) and that satisfied by $\sum_{k=1}^{M}kc_{k}(t)$ (Eq 5) differ by $\left(p(N)Mc_{M}+\frac{\gamma_{0}}{\Omega }c_{M}\right),$ and thus $N(t)\neq \sum_{k=1}^{M}kc_{k}.$ Thus, the very few numbers of clones of large sizes *k* > *M*, whose population is accounted for in Eq 1, are not accounted for fully in Eqs 2–4. This is especially salient in the *γ* → 0^+^ limit, in which the *N* > 0 fixed point is completely “missed” by the *c*_*k*_ = 0 solution to Eqs 2–4. Here, the *γ* → 0^+^ limit of the truncated system represents a singular limit where the only solution to Eqs 2–4, *c*_*k*_ → 0^+^, appears to violate the $N=\sum_{k=1}^{\infty }kc_{k}>0$ constraint at the stable fixed point.

Nonetheless, we can use the ODE system Eqs 2–4 to analyze the effects of changes in the thymic output rate *γ* provided we carefully use the solutions *c*_*k*_ and *N* that are consistent with the *N* = 0 and *N* > 0 fixed points. We denote the normal level of thymic export in an adult of a given age by *γ* = *γ*_0_. To represent diminished thymic activity during atrophy, we take *γ* ≪ *γ*_0_, or even *γ* = 0, depending on the severity of the atrophy. As the thymus is highly plastic, the changes in *γ* throughout the process of atrophy and recovery tend to be rapid. With this in mind, we model such cycles of disease with a piecewise ODE system. Specifically, let us observe a human’s response to disease-induced changes in thymic activity over some time interval *I* = [*t*_0_, *t*_*S* + 1_]. We assume that this individual’s thymic export rate undergoes *S* abrupt changes, at times *t*_1_, *t*_2_,..., *t*_*S*_, where *t*_0_ < *t*_1_ < *t*_2_ < … < *t*_*S*_ < *t*_*S* + 1_. Letting *I*_*i*_ = [*t*_*i*_, *t*_*i*+1_], so that $I=\overset{S}{\underset{i=0}{\cup }}I_{i},$ we assume that *γ* = *γ*_*i*_ ≥ 0, on *I*_*i*_. If the initial condition ${\left\{c_{k}\left(t_{0}\right)\right\}}_{k=1}^{M}$ represents the size of each *c*_*k*_ compartment at the start of the process, we then let ${\left\{c_{k}^{i}(t)\right\}}_{k=1}^{M}$ represent the solution of the ODE in Eqs 1–4, on *I*_*i*_, with *γ* = *γ*_*i*_ and initial condition ${\left\{c_{k}^{i}\left(t_{i}\right)\right\}}_{k=1}^{M}={\left\{c_{k}^{i-1}\left(t_{i}\right)\right\}}_{k=1}^{M},\;\text{for}\;i=1,2,\cdots ,S.$ Thus, the solution ${\left\{c_{k}^{i}(t)\right\}}_{k=1}^{M}$ represents the time evolution of the *c*_*k*_ compartments after a transition to a thymic activity level *γ*_*i*_. This is the most general description of our model; in practice, we will typically take *S* = 1, representing a single abrupt change in *γ*(*t*). Further descriptions of the piecewise ODE formulation specific to certain disease patterns and particular initial conditions are included in the relevant sections below.

Linear analysis of Eqs 2–4 will provide information on how the *clone counts* *c*_*k*_(*t*) evolve from their steady-state values after a small perturbation in the system (through changes in *γ*). The dynamics of *c*_*k*_ are not equivalent–but qualitatively related to–those of *n*_*i*_(*t*), the number of cells in clone *i*. For example, when large values of *n*_*i*_(*t*) increase, $c_{k\approx n_{i}}(t)$ decreases while $c_{k\approx n_{i}+1}(t)$ increases. Thus, increases in large *n*_*i*_ convect *c*_*k*_ forward, especially for larger *k*. As will be explicitly shown, since *c*_*k*_ is typically monotonically decreasing in *k*, the dynamics of small(large) clone populations are correlated with the dynamics of *c*_*k*_ for small(large) *k*.

## 3. Analysis for *γ* > 0 (functioning thymus)

We begin by studying the behavior of solutions of our ODE model under the assumption of a strictly positive thymic export rate, *γ* > 0. We perform an analysis of equilibrium solutions of Eqs 1–4 and their stability, and also compute an explicit solution in the infinite dimensional case that arises when *M* → ∞. At the beginning of sections 3.1 and 3.2 below, as well as in sections 4.1, 4.2 and 5, we focus on solutions over one individual interval in the piecewise formulation described in section 2 above. For simplicity when doing this, we omit the *i* notation that distinguishes the different subintervals, writing *γ* instead of *γ*_*i*_, etc. When the discussion returns to the full piecewise ODE, the *i* notation is reintroduced.

### 3.1 Analytic solution of the infinite dimensional system

We begin by computing analytic expressions for the solutions *c*_*k*_ of Eqs 1–4. If we take *M* → ∞ and consider instead the infinite dimensional system, the *c*_*k*_ compartments can be obtained through a generating function in a conjugate variable *q*, defined as

(6) $$Q(q,t)\equiv \sum_{k=0}^{\infty }c_{k}(t)q^{k},$$

Note that $\partial^{k}Q/{\partial q^{k}|}_{q=0}=k!c_{k},$ which allows us to extract *c*_*k*_ with *k* ≥ 0. In addition, the total population can also be recovered from the generating function via the expression $\partial Q/{\partial q|}_{q=1}=\sum_{k=0}^{\infty }kc_{k}=N.$

In order to derive an explicit form for *Q*(*q, t*), we assume that an explicit solution *N* = *N*(*t*) of Eq 1 can be found, so that we may write *p* and *μ* as functions of *t* (*p* = *p*(*t*), *μ* = *μ*(*t*)). By substituting Eqs 2 and 3 for d*c*_*k*_/d*t*, the time derivative of *Q* can be expressed as

(7) $$\frac{\partial Q}{\partial t}=\sum_{k=0}^{\infty }\frac{\text{d}c_{k}}{\text{d}t}q^{k}=(q-1)(p(t)q-\mu (t))\frac{\partial Q}{\partial q}+\frac{\gamma }{\Omega }(q-1)Q.$$

The above partial differential equation can be solved analytically along any characteristic curve *q*(*t*) defined by the solutions to $\frac{\text{d}q}{\text{d}t}=-(q-1)(p(t)q-\mu (t)):$

(8) $$q(t)=1+\frac{\left(1-q_{0}\right)A(t)}{\left(1-q_{0}\right)B(t)-1},$$

where *q*_0_ = *q*(0) and

(9) $$A(t)\equiv \exp\left(-\int_{0}^{t}(p(s)-\mu (s))\text{d}s\right)\;\text{and}\;B(t)\equiv \int_{0}^{t}p(s)A(s)\text{d}s.$$

Along each trajectory *q*(*t*), the generating function obeys $\frac{\text{d}Q}{\text{d}t}=-\frac{\gamma (t)}{\Omega }(1-q(t))Q$ and can be expressed as

(10) $$Q(q(t),t)=\sum_{k=0}^{\infty }c_{k}(0)q_{0}^{k}\exp\left(-\int_{0}^{t}\frac{\gamma (s)}{\Omega }(1-q(s))\text{d}s\right).$$

By allowing all possible initial values *q*_0_, we can express the full solution as

(11) $$Q(q,t)=\sum_{k=0}^{\infty }c_{k}(0){\left[1-\frac{1-q}{\left(1-q)B(t)+A(t\right)}\right]}^{k}\exp\left[-\int_{0}^{t}\text{d}s\frac{\gamma (s)}{\Omega }\frac{\left(1-q)A(s\right)}{\left(1-q)(B(t)-B(s))+A(t\right)}\right].$$

The solutions *c*_*k*_(*t*) can then be extracted from *Q*(*q, t*) by taking a series expansion of *Q*(*q, t*) and identifying coefficients with *c*_*k*_(*t*) according to Eq 6. These exact solutions *c*_*k*_(*t*) will be compared with our subsequent results derived from direct numerical solution of Eqs 1–4. By verifying that the time evolution of *c*_*k*_ under a finite dimensional formulation as in Eqs 2–4 is sufficiently close to that of *c*_*k*_ under the infinite dimensional formulation in Eq 11, we allow the infinite and finite dimensional systems to be used more or less interchangeably. The finite dimensional formulation has the advantage of not only admitting simple, explicit steady state solutions, but also rates of convergence to steady state.

### 3.2. Equilibrium solution and linearization

Returning to the truncated formulation (finite *M*), we now study the equilibrium solution that results when taking *γ* > 0 in Eqs 1–4. Denote such a generic equilibrium solution by ${\left\{c_{k}^{*}(\gamma )\right\}}_{k=1}^{M},N^{*}(\gamma ).$ For a given N^*^(*γ*), the $c_{k}^{*}(\gamma )$ have the form

(12) $$c_{1}^{*}(\gamma )=\gamma {\left[\sum_{i=1}^{M}\frac{\gamma /\Omega }{i!\mu {\left(N^{*}(\gamma )\right)}^{i-1}}\left(\prod_{j=1}^{i-1}\left[\frac{\gamma }{\Omega }+jp\left(N^{*}(\gamma )\right)\right]\right]+\mu \left(N^{*}(\gamma )\right)\right]}^{-1},$$

(13) $$c_{k}^{*}(\gamma )=\frac{c_{1}^{*}(\gamma )}{k!\mu {\left(N^{*}(\gamma )\right)}^{k-1}}\prod_{n=1}^{k-1}\left[\frac{\gamma }{\Omega }+np\left(N^{*}(\gamma )\right)\right].$$

In the discussion below, we will write $N^{*}(\gamma )\;\text{as}\;N^{*}\;\text{and}\;c_{k}^{*}(\gamma )\;\text{as}\;c_{k}^{*}$ for simplicity, unless desiring to emphasize the *γ*-dependence. To identify the stability of this equilibrium solution–and the rates of convergence of solutions to equilibria under the linearized model later on–we consider the linearization of the system around this generic equilibrium solution, represented by the $(M+1)\times (M+1)\;\text{matrix}\;L_{S}(L_{S}={\left(s_{ij}\right)}_{1\leq i,j\leq M+1}),$ with component *s*_*ij*_ given by

(14) $$\left\{\begin{array}{ll} -\left(\frac{2\gamma }{\Omega }\right)-\left(p\left(N^{*}\right)+\mu \left(N^{*}\right)\right), & \text{if}\;i=j=1\\ -\left(\frac{\gamma }{\Omega }\right)+2\mu \left(N^{*}\right), & \text{if}\;i=1,j=2\\ -\left(\frac{\gamma }{\Omega }\right), & \text{if}\;i=1;3\leq j\leq M\\ -\left(\frac{\gamma }{\Omega }\right)-i\left(p\left(N^{*}\right)+\mu \left(N^{*}\right)\right), & \text{if}\;i=j;2\leq j\leq M-1\\ -\left(\frac{\gamma }{\Omega }\right)+ip\left(N^{*}\right), & \text{if}\;i=j+1;1\leq j\leq M-1\\ -M\left(p\left(N^{*}\right)+\mu \left(N^{*}\right)\right), & \text{if}\;i=j=M\\ (i+1)\mu \left(N^{*}\right), & \text{if}\;i=j-1;\;2\leq j\leq M\\ p^{\prime }\left(N^{*}\right)[(j-1)c_{j-1}^{*}-jc_{j}^{*}]+\mu^{\prime }\left(N^{*}\right)[(j+1)c_{j+1}^{*}-jc_{j}^{*}], & \text{if}\;i=M+1;1\leq j\leq M-1\\ p^{\prime }\left(N^{*}\right)(M-1)c_{M-1}^{*}-\mu^{\prime }\left(N^{*}\right)Mc_{M}^{*}, & \text{if}\;i=M+1;j=M\\ p^{\prime }\left(N^{*}\right)N^{*}+p\left(N^{*}\right)-\mu^{\prime }\left(N^{*}\right)N^{*}-\mu \left(N^{*}\right), & \text{if}\;i=j=M+1\\ 0, & \text{otherwise}. \end{array}\right\}$$

For clarity, an example of the matrix *L*_*S*_ with *M* = 4 is

$$L_{s}=\left(\begin{array}{ccccc} -\frac{2\gamma }{\Omega }-\left(p\left(N^{*}\right)+\mu \left(N^{*}\right)\right) & -\frac{\gamma }{\Omega }+2\mu \left(N^{*}\right) & -\frac{\gamma }{\Omega } & -\frac{\gamma }{\Omega } & -p^{\prime }\left(N^{*}\right)c_{i}^{*}\\ \frac{\gamma }{\Omega }+p\left(N^{*}\right) & -\frac{\gamma }{\Omega }-2\left(p\left(N^{*}\right)+\mu \left(N^{*}\right)\right) & 3\mu \left(N^{*}\right) & 0 & p^{\prime }\left(N^{*}\right)\left[c_{1}^{*}-2c_{2}^{*}\right]+\mu^{\prime }\left(N^{*}\right)[3c_{3}^{*}-2c_{2}^{*}]\\ 0 & \frac{\gamma }{2}+2p\left(N^{*}\right) & -\frac{\gamma }{\Omega }-3\left(p\left(N^{*}\right)+\mu \left(N^{*}\right)\right) & 4\mu \left(N^{*}\right) & p^{\prime }\left(N^{*}\right)\left[2c_{2}^{*}-3c_{3}^{*}\right]+\mu^{\prime }(N^{*})[4c_{4}^{*}-3c_{3}^{*}]\\ 0 & 0 & \frac{\gamma }{\Omega }+3p\left(N^{*}\right) & -4\mu \left(N^{*}\right) & 3p^{\prime }\left(N^{*}\right)c_{3}^{*}-4\mu \left(N^{*}\right)c_{4}^{*}\\ 0 & 0 & 0 & 0 & p^{\prime }\left(N^{*}\right)N^{*}+p\left(N^{*}\right)-\mu^{\prime }\left(N^{*}\right)N^{*}-\mu \left(N^{*}\right) \end{array}\right)$$

We now apply a simplifying assumption to the matrix *L*_*s*_ to analytically compute its eigenvalues more easily. In general, using previous estimates for *γ* [55] and Ω [56,57], $\frac{\gamma }{\Omega }\sim 10^{-8}-10^{-6},\;\text{and}\;p\left(N^{*}\right),\mu \left(N^{*}\right)\sim 10^{-1}$ (when rates are measured in units of year^−1^), thus, we assume $\frac{\gamma }{\Omega }\ll p\left(N^{*}\right),\mu \left(N^{*}\right)$ and define a new matrix $L_{\tilde{S}}$ which is *L*_*S*_ but with $\frac{\gamma }{\Omega }=0$ only in elements in which it appears explicitly. $L_{\tilde{S}}$ is defined by Eq 14 with γ/Ω in the first five terms set to zero, but with N* determined under the appropriate general immigration rate *γ* ≥ 0.

Numerical computation confirms that the eigenvalues of the new matrix $L_{\tilde{S}}$ are essentially identical to those of the original matrix $L_{S}\left(\frac{\gamma }{\Omega }\ll 1\right),$ validating our assumption that the term $\frac{\gamma }{\Omega }$ may be neglected in *L*_*S*_. Note that this assumption does not cause us to omit the constant *γ* from the linearization matrix *L*_*S*_ entirely, as the steady state values $N^{*},c_{k}^{*}$ depend on *γ*. Denote by $\lambda_{k}^{S}\;\text{for}\;k=1,2,\cdots ,M+1$ the eigenvalues of $L_{\tilde{S}}$, and note that that the entry ${\tilde{s}}_{\left(M+1,M+1\right)}={\frac{\text{d}N}{\text{d}t}|}_{N=N^{*}}=p^{\prime }\left(N^{*}\right)N^{*}+p\left(N^{*}\right)-\mu^{\prime }\left(N^{*}\right)N^{*}-\mu \left(N^{*}\right)$ is an eigenvalue. We denote this eigenvalue by $\lambda_{M+1}^{S}.$ Since N^*^ is the stable equilibrium solution of Eq 1, we assume that $\lambda_{M+1}^{S}<0.$ As shown in [58], the eigenvalues of $L_{\tilde{S}}$ all have strictly negative real part, as do those of *L*_*S*_. The eigenvalues of the (M+1)×(M+1) minor of *L*_*S*_ can be approximated by ${\tilde{\lambda }}_{k}^{S}=k\left(p\left(N^{*}\right)-\mu \left(N^{*}\right)\right),$ with corresponding eigenvectors ${\tilde{y}}_{k}$ shown in the following proposition:

#### Proposition 3.1.

*If* $p\left(N^{*}\right)-\mu \left(N^{*}\right)<0,$ *the eigenvalues* ${\left\{\lambda_{k}^{S}\right\}}_{k=1,\ldots ,M}$ *of the matrix* $L_{\tilde{S}}$ *are well approximated by the terms* ${\tilde{\lambda }}_{k}^{S}=k\left(p\left(N^{*}\right)-\mu \left(N^{*}\right)\right),$ *in the sense that there exist vectors* ${\tilde{y}}_{k}$ *such that* $\left\|\left(L_{\tilde{S}}-{\tilde{\lambda }}_{k}^{S}I\right){\tilde{y}}_{k}\right\|\to 0\;\text{as}\;M\to \infty .$

*Proof.* We begin by assuming that the terms ${\tilde{\lambda }}_{k}^{S}=k\left(p\left(N^{*}\right)-\mu \left(N^{*}\right)\right)$ are themselves eigenvalues of $L_{\tilde{S}}$, and search for their corresponding eigenvectors, ${\tilde{y}}_{k}=\left({\tilde{y}}_{k}^{1},{\tilde{y}}_{k}^{2},\cdots ,{\tilde{y}}_{k}^{M},0\right).$ Choosing ${\tilde{y}}_{k}^{1}=1,$ we then choose ${\tilde{y}}_{k}^{i}$ for i=2,…,M inductively so as to force the *i*-th component of the residual vector, which we denote by ${\left[\left(L_{\tilde{S}}-{\tilde{\lambda }}_{k}^{S}I\right){\tilde{y}}_{k}\right]}_{i},$ to equal zero for i=1,2,⋯,M−1. We then verify that ${\left[\left(L_{\tilde{S}}-{\tilde{\lambda }}_{k}^{S}I\right){\tilde{y}}_{k}\right]}_{M}\to 0\;\text{as}\;M\to \infty ,$ so that for $M\gg 1,\left\|\left(L_{\tilde{s}}-{\tilde{\lambda }}_{k}^{S}I\right){\tilde{y}}_{k}\right\|\approx 0,$ where ||·|| is any *p*-norm. (Trivially, ${\left[\left(L_{\tilde{S}}-{\tilde{\lambda }}_{k}^{S}I\right){\tilde{y}}_{k}\right]}_{M+1}=0.)$ We first note that the components ${\tilde{y}}_{k}^{1},\cdots ,{\tilde{y}}_{k}^{M}$ of the approximate eigenvector ${\tilde{y}}_{k}$ corresponding to eigenvalue ${\tilde{\lambda }}_{k}^{S}$ are defined by the recurrence relation,

(15) $$i{\tilde{y}}_{k}^{i}=\left[\left(i+(k-1))\left(\frac{p\left(N^{*}\right)}{\mu \left(N^{*}\right)}\right)+(i-(k+1)\right)\right]{\tilde{y}}_{k}^{i-1}-[i-2]\left(\frac{p\left(N^{*}\right)}{\mu \left(N^{*}\right)}\right){\tilde{y}}_{k}^{i-2}.$$

The solution of this recurrence relation is then

(16) $${\tilde{y}}_{k}^{i}=\sum_{n=1}^{k}\frac{\left[\prod_{j=1}^{n-1}\left(i-j\right)\right]\left[\prod_{j=1}^{k-n}\left(i+j\right)\right]}{k{\left(-1\right)}^{n-1}(n-1)!(k-n)!}{\left(\frac{p\left(N^{*}\right)}{\mu \left(N^{*}\right)}\right)}^{i-n},$$

where we let $\prod_{j=1}^{0}\left(i\pm j\right)=1,$ whenever such a term appears in the above sum. (This is verified in Appendix 6.) As previously mentioned, ${\left[\left(L_{\tilde{S}}-{\tilde{\lambda }}_{k}^{S}I\right){\tilde{y}}_{k}\right]}_{i}=0\;\text{for}\;i=1,2,\cdots ,M-1.$ We now compute ${\left[\left(L_{\tilde{S}}-{\tilde{\lambda }}_{k}^{S}I\right){\tilde{y}}_{k}\right]}_{M},$ obtaining

(17) $${\left[\left(L_{\tilde{S}}-{\tilde{\lambda }}_{k}^{S}I\right){\tilde{y}}_{k}\right]}_{M}=(M-1)p\left(N^{*}\right){\tilde{y}}_{k}^{M-1}-M\mu \left(N^{*}\right){\tilde{y}}_{k}^{M}\;\;=p\left(N^{*}\right)\sum_{n=1}^{k}\frac{\left[\prod_{j=0}^{n-1}\left(M-1-j\right)\right]\left[\prod_{j=1}^{k-n}\left(M-1+j\right)\right]}{k{\left(-1\right)}^{n-1}(n-1)!(k-n)!}{\left(\frac{p\left(N^{*}\right)}{\mu \left(N^{*}\right)}\right)}^{M-1-n}\;\;-\mu \left(N^{*}\right)\sum_{n=1}^{k}\frac{\left[\prod_{j=0}^{n-1}\left(M-j\right)\right]\left[\prod_{j=1}^{k-n}\left(M+j\right)\right]}{k{\left(-1\right)}^{n-1}(n-1)!(k-n)!}{\left(\frac{p\left(N^{*}\right)}{\mu \left(N^{*}\right)}\right)}^{M-n}.$$

Each term in the sum above has the form $p_{k}(M)a^{M},\;\text{where}\;p_{k}(M)$ is a polynomial of degree *k* in the variable *M*, and *a* = *p*(N*)/*μ*(N*). Recalling that *p*(N*)/*μ*(N*) < 1, then $\lim_{M\to \infty }p_{k}(M)a^{M}=0,$ so that at large values of $M,{\left[\left(L_{\tilde{S}}-{\tilde{\lambda }}_{k}^{S}I\right){\tilde{y}}_{k}\right]}_{M}\approx 0.$ This demonstrates that ${\tilde{\lambda }}_{k}^{S}$ may be regarded as an approximation to the true eigenvalue $\lambda_{k}^{S},$ assuming that the eigenvalues of $L_{\tilde{S}}$ are stable under small perturbations. □

**Figure 1.**
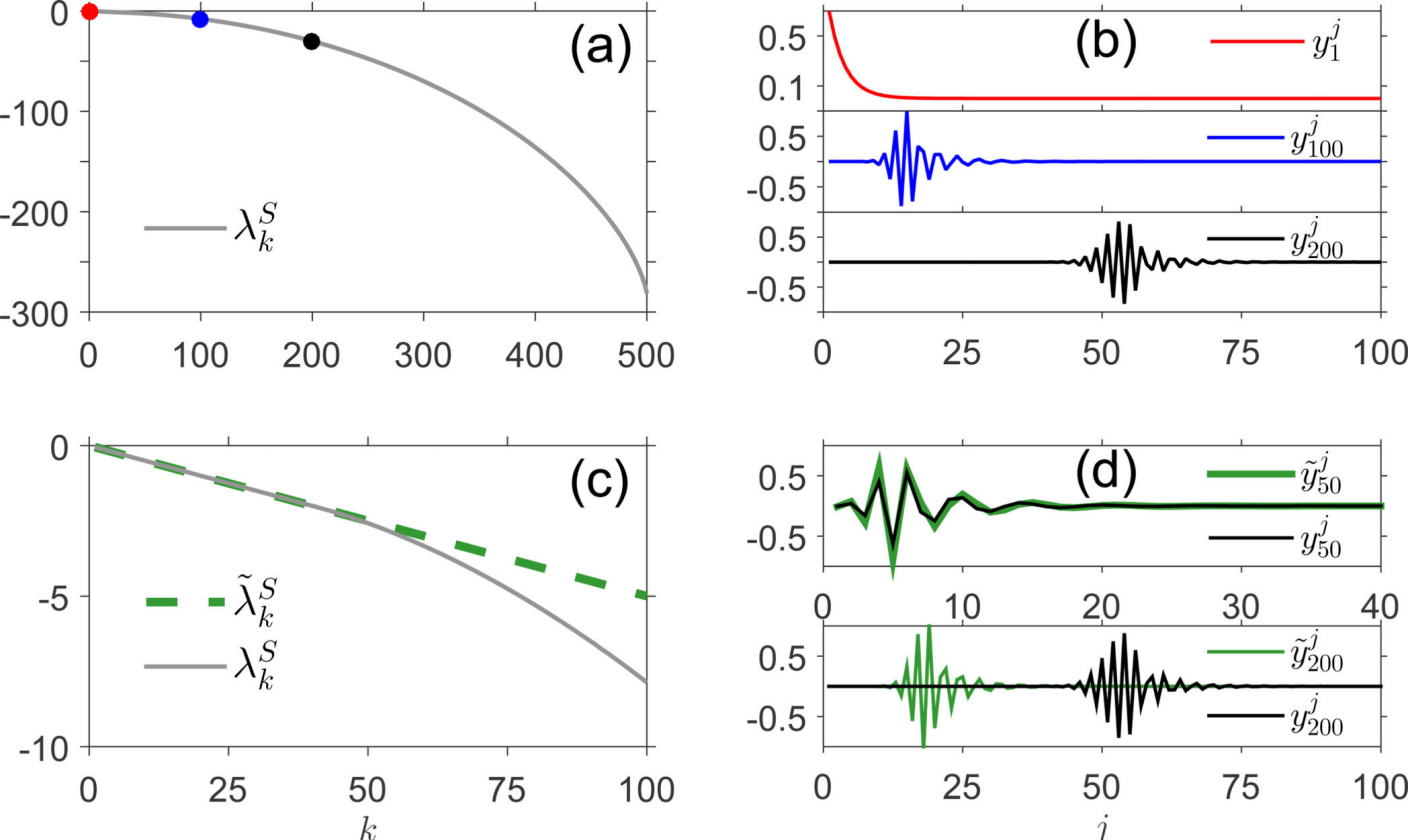
Eigenvalues and eigenvectors of $L_{\tilde{S}},\gamma >0.$ Here, and in all subsequent evaluations, we parameterize our model using values in a range based qualitatively on human data, where the unit of rates are expressed in 1/year [55,59,60]. However, the model can have arbitrary units in general cases. (a) Numerically computed eigenvalue spectrum of the matrix $L_{\tilde{S}},$ with $p\left(N^{*}\right)=0.12,\mu \left(N^{*}\right)=0.17\;\text{and}\;M=500$. Dots identify the locations of the eigenvalues $\lambda_{1}^{S}(\text{red}),\lambda_{100}^{S}(\text{blue}),\lambda_{200}^{S}(\text{black}).$ (b) First 100 components $\left({\tilde{y}}_{k}^{1},\cdots ,{\tilde{y}}_{k}^{100}\right)$ of the eigenvectors with indices *k* = 1, 100, 200, the eigenvalues corresponding to which are marked on the spectral curve in (a). (c) Comparison of true eigenvalues $\left(\lambda_{k}^{S}\right)$ and approximate eigenvalues $\left({\tilde{\lambda }}_{k}^{S}\right)\;\text{for}\;k=1,\cdots ,100$. Approximation is strong for k≲50. (d) (Top) comparison of ${\tilde{y}}_{50}^{j}\;\text{and}\;y_{50}^{j}\;\text{for}\;j=1,\cdots ,40,$ showing that the approximation is strong. (Bottom) comparison of ${\tilde{y}}_{200}^{j}\;\text{and}\;y_{200}^{j}\;\text{for}\;j=1,\cdots ,100,$ showing that the accuracy of the approximation breaks down, but the qualitative behavior of $y_{200}^{j}$ is captured in ${\tilde{y}}_{200}^{j}.$

The solutions ${\tilde{y}}_{k}^{i},$ as functions of *i* for fixed *k*, are characterized by patterns of oscillatory behavior, as shown in Figure 1, which depicts numerical solutions of true and approximate eigenvalues and eigenvectors of $L_{\tilde{S}}.$ We choose $p\left(N^{*}\right)\;\text{and}\;\mu \left(N^{*}\right)\;\text{to}\;\text{satisfy}\;p\left(N^{*}\right)<\mu \left(N^{*}\right),$ so that at homeostatic population levels, the death rate exceeds the proliferation rate, preventing exponential growth of the population.

Figure 1a presents a sample plot of the eigenvalue spectrum $\lambda_{k}^{S}$ in the case *M* = 500; on the spectral curve, several eigenvalues are marked, for which the first 100 components of the corresponding eigenvectors are plotted in Figure 1b. As discussed previously, all eigenvalues are negative. The eigenvector plots in Figure 1b indicate that as *k* increases, the oscillatory “mass” of the corresponding eigenvectors occurs at increasingly large values of *j*. Figure 1c presents a comparison of the true eigenvalue spectrum $\left(\lambda_{k}^{S}\right)$ and the approximate eigenvalue spectrum $\left({\tilde{\lambda }}_{k}^{S}\right)$ of the matrix $L_{\tilde{S}}.$ The eigenvalue approximation is very strong for 1≲k≲M/10, and this remains true as *M* varies. The quantities ${\tilde{\lambda }}_{k}^{S}=k\left(p\left(N^{*}\right)-\mu \left(N^{*}\right)\right)$ over-approximate the $\lambda_{k}^{S}\;\text{for}\;k≳M/10$. Figure 1d depicts a comparison of $y_{k}\;\text{and}\;{\tilde{y}}_{k}$ for two values of *k*, one below the crossover ~ *M*/10 (*k* = 50, top) and one above *M*/10 (*k* = 200, bottom). As expected, the eigenvector approximation is accurate precisely when the corresponding eigenvalue approximation is accurate. Even for k≳M/10, the approximate eigenvectors ${\tilde{y}}_{k}$ present an appearance similar to that of the ${\tilde{y}}_{k}$ for smaller *k*. The diminished accuracy of the eigenvalue/eigenvector pairs at higher *k* (for fixed *M*) is attributable to the slower convergence of ${\left[\left(L_{S}-{\tilde{\lambda }}_{k}^{S}I\right){\tilde{y}}_{k}\right]}_{M}$ to 0 as *M* → ∞ for larger *k*, which is immediately apparent from the form in Eq 17. For a system of a fixed dimension *M*, increasing *μ*(N*) relative to *p*(N*) causes the approximate eigenvalues ${\tilde{\lambda }}_{k}^{S}$ to become increasingly accurate at larger *k*, clearly due to the quicker convergence of the residual quantity $\left\|\left(L_{\tilde{S}}-{\tilde{\lambda }}_{k}^{S}I\right){\tilde{y}}_{k}\right\|\;\text{to}\;0$ when *p*(N*)/*μ*(N*) ≪ 1, as indicated by Eq 16.

**Figure 2.**
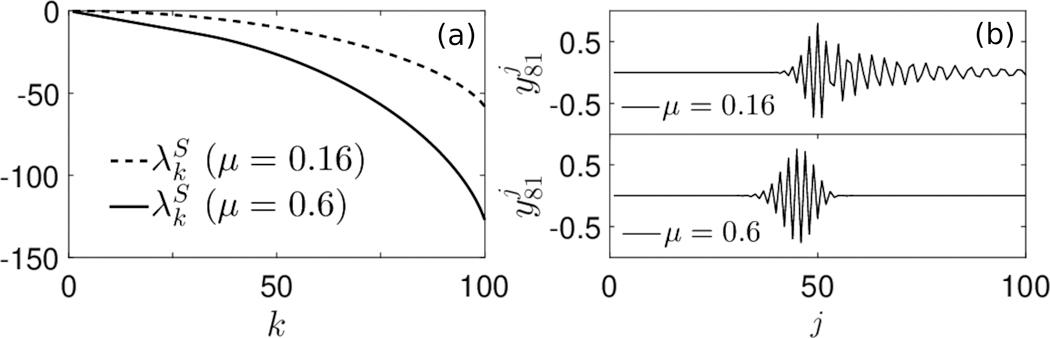
Eigenvalues and eigenvectors of $L_{\tilde{S}},\gamma >0,$ varying *μ*. (a) Numerically computed eigenvalues, $\lambda_{k}^{S},\;\text{for}\;k=1,\cdots ,100,$ when *μ*(N*) = 0.16 and *μ*(N*) = 0.6. In both cases, *p*(N*) = 0.15, *M* = 100. (b) Numerically computed eigenvectors *y*_81_.

Increases to *μ*(N*) relative to *p*(N*) also cause an intensified dampening of the oscillations in the eigenvector ${\tilde{y}}_{k}$ at lower components *j*, which creates the illusion of the oscillatory mass shifting to the left as *μ*(N*) increases for fixed *p*(N*), as in Figure 2b. At the same time, the entire eigenvalue spectrum becomes more negative as *μ*(N*) increases, as indicated in Figure 2a, so that increases to the death rate at homeostatic levels indicate much faster convergence to equilibrium of all *c*_*k*_ compartments.

### 3.3. Behavior of the linearized and fully nonlinear systems

This section addresses the convergence behavior of solutions in the presence of a positive thymic export rate *γ* > 0. This situation represents a functioning thymus, with the possibility for many different levels of functionality, ranging from total health $\left(\mathrm{high}\;\gamma \sim \gamma_{0}\right)$ to dramatically diminished functionality (low *γ*). It could also represent a new thymic export rate after transplant of thymic tissue, as studied in the context of DiGeorge’s syndrome in Ciupe *et al*. [40]. In this case, we determined that for each equilibrium solution of Eq 1, the system has an equilibrium solution given by Eqs 12 and 13. If the steady state solution N^*^(*γ*) > 0 of Eq 1 represents a stable fixed point, the corresponding equilibrium $c_{k}^{*}(\gamma )$ will also be stable (typical regulated forms of proliferation and death, *p*(*N*) and *μ*(*N*), tend to result in one positive stable equilibrium solution in Eq 1). If for some *i*, *γ*_*i*_ > 0, the solution ${\left\{c_{k}^{i}(t)\right\}}_{k=1}^{M},N^{i}(t)\;\text{satisfies}\;c_{k}^{i}(t)\to c_{k}^{*}\left(\gamma_{i}\right),N^{i}(t)\to N^{*}\left(\gamma_{i}\right).$ The convergence of the total population $N^{i}(t)\to N^{*}$ occurs at rate $p^{\prime }\left(N^{*}\right)N^{*}+p\left(N^{*}\right)-\mu^{\prime }\left(N^{*}\right)N^{*}-\mu \left(N^{*}\right).$ Based on the eigenvalues and eigenvectors of the approximate linearization, $L_{\tilde{S}},$ of Eqs 1–4 around this equilibrium, we can formally construct approximations to the time-dependent solutions for *c*_*k*_(*t*).

In the fully nonlinear system, however, the linearized eigenvalues provide only a *priori* rates of convergence of solution trajectories initialized near equilibrium. The accuracy of the eigenvalues in providing convergence rates of solutions depends on the initial conditions. If the initial conditions, $c_{k}^{i}\left(t_{i}\right),N^{i}\left(t_{i}\right),\;\text{satisfy}\;c_{k}^{i}\left(t_{i}\right)\sim c_{k}^{*}\left(\gamma_{i}\right),N^{i}\left(t_{i}\right)\sim N^{*}\left(\gamma_{i}\right),$ then the solutions begin near the stable equilibrium, and the eigenvalues provide accurate rates of convergence. If the initial conditions are far from equilibrium, the eigenvalues may not provide accurate rates of convergence of the entire solution trajectory. When trajectories are far from equilibrium at time *t*_*i*_, further information about the speed of convergence can be discerned from the relationship between $p\left(N^{i}\left(t_{i}\right)\right)-\mu \left(N^{i}\left(t_{i}\right)\right)\;\text{and}\;p\left(N^{*}\left(\gamma_{i}\right)\right)-\mu \left(N^{*}\left(\gamma_{i}\right)\right),$ the disparity in proliferation and death rates at the starting and terminal population levels. If these quantities differ significantly, solution trajectories are generally characterized by a transient period of fast convergence, which carries the trajectory close enough to the stable equilibrium that convergence rates from then on are dictated by the linearized eigenvalues. For example, assume that an abrupt drop in thymic productivity occurs at $t_{i},\;\text{so that}\;\gamma_{i}<\gamma_{i-1}.$ As the naive T cell population evolves from $N^{i}\left(t_{i}\right)\;\text{to}\;N^{*}\left(\gamma_{i}\right),$ for which $0>p\left(N^{*}\left(\gamma_{i}\right)\right)-\mu \left(N^{*}\left(\gamma_{i}\right)\right)>p\left(N^{i}\left(t_{i}\right)\right)-\mu \left(N^{i}\left(t_{i}\right)\right),$ the T cell pool will experience a brief period of higher cellular death. As *N*(*t*) approaches $N^{*}\left(\gamma_{i}\right),$ the convergence rates correspond to the eigenvalues found from the linearized approximation.

## 4. Analysis for *γ* = 0 (full thymic cessation)

We now proceed to study the system after thymic export is shut off (*γ* = 0). As in section 3 above, we compute equilibrium solutions of the truncated system (finite *M*) that arise when *γ* = 0, and identify the rates of convergence of the different *c*_*k*_(*t*) to equilibrium under the linearized model. We also take *M* → ∞ and consider explicit solutions of the infinite-dimensional system.

### 4.1 Analytic solutions

In the *γ* = 0 case, the solution *c*_*k*_(*t*) for *M* → ∞ can be readily expressed using the method of characteristics. By using the generating function *Q*(*q*, *t*) defined in Eq 11 and taking the *k*-th order derivative of *Q* with respect to *q* at *q* = 0, we find

(18) $$c_{k}(t)={\left[\frac{B(t)}{A(t)+B(t)}\right]}^{k}\sum_{i=0}^{\infty }c_{i}(0)\sum_{j=0}^{i}\left(\begin{array}{c} i\\ j \end{array}\right)\left(\begin{array}{c} k+j-1\\ k \end{array}\right){\left(1-\frac{1}{B(t)}\right)}^{i-j}{\left(\frac{A(t)}{B(t)(A(t)+B(t))}\right)}^{j}$$

and $N(t)=A^{-1}(t)\sum_{k=0}^{\infty }kc_{k}(0).$ Note that for depleted initial conditions $c_{0}(0)=\Omega \;\text{and}\;c_{k}(0)=0\;\text{for}\;k\geq 1,$ Eq 18 leads to $c_{0}(t)=\Omega \;\text{and}\;c_{k}(t)=0\;\text{for}\;k\geq 1$ at all times. Indeed, the T cell pool is expected to remain empty since there is no thymic export.

**Figure 3.**
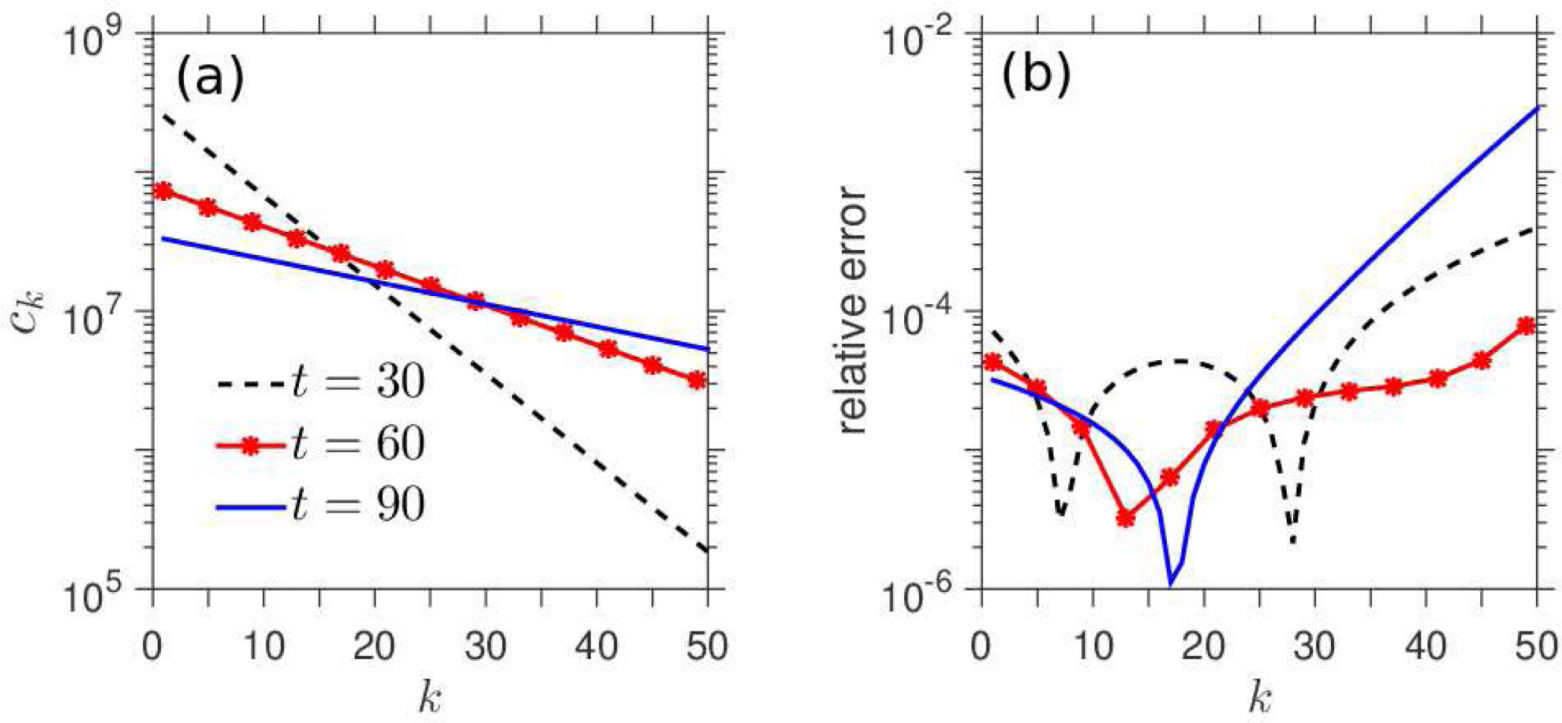
Computation of *c*_*k*_ from method of characteristics, comparison with truncated system. (a) Plots of $c_{k}\;\text{for}\;k=1,2,\cdots ,50,$ at times *t* = 30, 60, 90. Solutions *c*_*k*_ were computed numerically from the analytic method described in 4.1, based on the infinite-dimensional system. As a function of *k, c*_*k*_ presents as linear on a logarithmic scale. (b) Relative error, $\left|c_{k}-y_{k}\right|/c_{k}\;\text{for}\;k=1,2,\cdots ,50,$ at times *t* = 30, 60, 90, where *c*_*k*_ denotes the solutions depicted in (a), and *y*_*k*_ denotes the numerically computed solutions of the truncated system in Eqs 2–4. From (b), the disparity between the solutions of the infinite dimensional systems (*c*_*k*_) and the finite dimensional truncated systems (*y*_*k*_) is negligible, validating our decision to use them interchangeably. Coefficient functions: $p(N)=p_{0}>0,\mu (N)=\mu_{0}+\mu_{1}\left(N^{2}/\left(N^{2}+K^{2}\right)\right).$ Parameter values: $p_{0}=0.18,\mu_{0}=0.17,\mu_{1}=0.04,K=10^{10},\Omega =10^{16},M=100.$ Initial condition: $c_{0}(0)=10^{16}-10^{10},c_{1}(0)=10^{10},c_{k}(0)=0\;\text{for}\;k\geq 2$.

Figure 3a depicts a numerical computation of the solution, *c*_*k*_, of the infinite-dimensional formulation, obtained from the generating function. We include values of *c*_*k*_ for *k* = 1, 2,…, 50 at times *t* = 30, 60, 90. As a function of *k*, the *c*_*k*_ present as linear on a logarithmic scale, as expected. To compare the infinite dimensional system with the truncated system, we also compute solutions, *y*_*k*_, of the truncated Eqs 2–4 (not pictured). Figure 3b depicts the relative error, $\left|c_{k}-y_{k}\right|/c_{k}.$ As we see, the error is several orders of magnitude smaller than *c*_*k*_, *y*_*k*_ themselves at each of the times *t* = 30, 60, 90, indicating that the numerical solution of the truncated system Eqs 2–4 is accurately described by the exact method-of-characteristics solution and that the infinite- and finite- dimensional systems may be used more or less interchangeably.

### 4.2 Equilibrium solutions and linearization

We now investigate the solution *c*_*k*_(*t*) near the fixed points that arise when we take *γ* = 0 in Eqs 1–4. In this case, the system has two possible equilibrium solutions. Denoting generic equilibrium solutions by ${\left\{c_{k}^{*}\right\}}_{k=1}^{M}\;\mathrm{and}\;N^{*},$ the unstable solution is $c_{k}^{*}=0$ for all *k* ≥ 1 and N* = 0, and the asymptotically stable solution is $c_{k}^{*}=0$ for all 1 ≤ *k* ≤ *M*. However, in the stable state we define $N^{*}=\tilde{N}>0,$ where $\tilde{N}$ satisfies $p(\tilde{N})=\mu (\tilde{N}).$ To verify the stability of these solutions, we consider the linearization of the system around this equilibrium, which is represented by the (M+1)×(M+1) matrix we call $L_{U}\left(L_{U}={\left(u_{ij}\right)}_{1\leq i,j\leq M+1}\right).$ The components *u*_*ij*_ of *L*_*U*_ are given explicitly by:

(19) $$\left\{\begin{array}{ll} -j\left(p\left(N^{*}\right)+\mu \left(N^{*}\right)\right), & \text{if}\;i=j\leq M-1\\ -M\mu \left(N^{*}\right), & \text{if}\;i=j=M\\ j\mu \left(N^{*}\right), & \text{if}\;i=j-1;2\leq j\leq M\\ jp\left(N^{*}\right), & \text{if}\;i=j+1;1\leq j\leq M-1\\ p^{\prime }\left(N^{*}\right)N^{*}+p\left(N^{*}\right)-\mu^{\prime }\left(N^{*}\right)N^{*}-\mu \left(N^{*}\right), & \text{if}\;i=j=M+1\\ 0. & \text{otherwise} \end{array}\right\}$$

As before, $u_{\left(M+1),(M+1\right)}=p^{\prime }\left(N^{*}\right)N^{*}+p\left(N^{*}\right)-\mu^{\prime }\left(N^{*}\right)N^{*}-\mu \left(N^{*}\right)$ is an eigenvalue with eigenvector (0,...,0,1), and the remaining eigenvalues are those of the (M+1)×(M+1) minor of *L*_*U*_. All eigenvalues of the (*M* + 1) × (*M* + 1) minor have negative real part, and the stability of an equilibrium solution depends on the sign of $U_{\left(M+1),(M+1\right)}.$ If N* = 0, then $u_{\left(M+1),(M+1\right)}=p(0)-\mu (0)>0,$ as described previously, and the equilibrium $c_{k}^{*}=0,N^{*}=0$ is unstable. On the other hand, if $N^{*}=\tilde{N}\;\text{with}\;p(\tilde{N})=\mu (\tilde{N}),$ then N* represents a positive, homeostatic cell count, and $u_{\left(M+1),(M+1\right)}=\left(p^{\prime }\left(N^{*}\right)-\mu^{\prime }\left(N^{*}\right)\right)N^{*}<0,$ as *p*(*N*), *μ*(*N*) are assumed to be non-increasing and non-decreasing, respectively. Therefore, we have that $c_{k}^{*}=0\;\text{and}\;N^{*}=\tilde{N}>0$ is a stable equilibrium solution.

If *γ* = 0, the solution ${\left\{c_{k}(t)\right\}}_{k=1}^{M},N(t),$ will evolve away from the unstable equilibrium $c_{k}^{*}=0,N^{*}=0$ and towards the equilibrium $c_{k}^{*}=0,N^{*}=\tilde{N}>0.$ In this instance, the pool of low-population clones is eradicated due to lack of thymic productivity, and the high lymphopenic proliferation rate pushes existent clones past the truncation threshold *M*, where they are no longer accounted for in *c*_*k*_ but are accounted for in *N*, causing *N*(*t*) → N* despite the fact that *c*_*k*_(*t*) → 0 for all *k*. As before, we wish to explore further the rates at which individual functions *c*_*k*_ diverge from the unstable fixed point towards the stable one under the linearized and fully nonlinear models. To this end, we study the eigenvalues of the linearization matrix, *L*_*U*_, evaluated at the two equilibria.

First, consider the eigenvalues of *L*_*U*_ evaluated at the unstable equilibrium. In this case, we assume *p*(0) > *μ*(0), as described earlier. Without thymic export, new clones are not generated in the periphery, and existent clones expand due to the high proliferation rate. Under the dynamics described by Eqs 2–4, clones quickly expand beyond the small-*k* compartments and get “caught” at the boundary at size *M*, before depleting due to the slow death-induced passage of single cell clones through the boundary at *k* = 1. According to Eq 1, the total cell population reaches a natural homeostatic level through peripheral maintenance alone. To investigate the rates at which these processes occur under the linearized model, we derive approximations to the dominant eigenvalues of *L*_U_. Under the assumption that *p*(0) > *μ*(0), we denote the true eigenvalues of $L_{U}\;\text{by}\;\lambda_{k}^{U}$ for *k* = 0, 1,…*M*, with corresponding eigenvectors $z_{k}=\left(z_{k}^{M},z_{k}^{M-1},\cdots ,z_{k}^{1},z_{k}^{0}\right)$ (note that we have reversed the index ordering here). Assign to the eigenvalue $u_{\left(M+1),(M+1\right)}=p(0)-\mu (0)$ the label $\lambda_{M}^{U},$ and to its eigenvector (0,...,0,1) the label *z*_*M*_. What remains is to find approximations to the other *M* eigenvalues of *L*_*U*_, which are precisely the eigenvalues of the (*M* + 1) × (*M* + 1) minor. For *k* = 0,1,...,*M* − 1, denote the approximation to the eigenvalue $\lambda_{k}^{U}\;\text{by}\;{\tilde{\lambda }}_{k}^{U},$ and the approximation to the eigenvector $z_{k}\;\text{by}\;{\tilde{z}}_{k}=\left({\tilde{z}}_{k}^{M},{\tilde{z}}_{k}^{M-1},\cdots ,{\tilde{z}}_{k}^{1},0\right).$ We begin by establishing that the eigenvalue of the (*M* + 1) × (*M* + 1) minor with the smallest magnitude, $\lambda_{0}^{U},$ is well approximated by ${\tilde{\lambda }}_{0}^{U}=0.$

#### Proposition 4.1.

*The eigenvalue of L_U_ of smallest magnitude*, $\lambda_{0}^{U},$ *is well approximated by* ${\tilde{\lambda }}_{0}^{U}=0,$ *in the sense that there exists a vector* ${\tilde{z}}_{0}=\left({\tilde{z}}_{0}^{M},{\tilde{z}}_{0}^{M-1},\cdots ,{\tilde{z}}_{0}^{2},{\tilde{z}}_{0}^{1},0\right)\;such\;that\;\left\|\left(L_{U}-{\tilde{\lambda }}_{0}^{U}I\right){\tilde{z}}_{0}\right\|\to 0\;as\;M\to \infty .$

*Proof.* (Note: The components of ${\tilde{z}}_{0}$ are written above in “descending” order for notational convenience, as this reflects the order in which they will be chosen recursively below. The *i*-th component from the left of ${\tilde{z}}_{0}$, denoted explicitly by ${\tilde{z}}_{0}^{M-i+1},$ still corresponds to the function *c*_*i*_.) We begin by considering the matrix $\left(L_{U}-{\tilde{\lambda }}_{0}^{U}I\right)=L_{U}$ and searching for an appropriate eigenvector, ${\tilde{z}}_{0}.$ We define ${\tilde{z}}_{0}^{1}=1,$ and once again choose the components ${\tilde{z}}_{0}^{i}$ inductively via a three-term recurrence relation so as to force the *i*-th component of $\left(L_{U}-{\tilde{\lambda }}_{0}^{U}I\right){\tilde{z}}_{0}.$ which we denote as before by ${\left[\left(L_{U}-{\tilde{\lambda }}_{0}^{U}I\right){\tilde{z}}_{0}\right]}_{i},$ to satisfy ${\left[\left(L_{U}-{\tilde{\lambda }}_{0}^{U}I\right){\tilde{z}}_{0}\right]}_{i}=0$ for *i* = 2, 3, …, *M* + 1. While ${\left[\left(L_{U}-{\tilde{\lambda }}_{0}^{U}I\right){\tilde{z}}_{0}\right]}_{1}\neq 0,$ we show that ${\left[\left(L_{U}-{\tilde{\lambda }}_{0}^{U}I\right){\tilde{z}}_{0}\right]}_{1}\to 0\;\text{as}\;M\to \infty ,$ to, so that ${\tilde{z}}_{0}$ may be regarded formally as an “approximate” eigenvector corresponding to the approximate eigenvalue ${\tilde{\lambda }}_{0}^{U}.$

Defining ${\tilde{z}}_{0}^{1}=1\;\text{and}\;{\tilde{z}}_{0}^{2}=\frac{M\mu (0)}{\left(M-1)p(0\right)},\;\text{we let}\;{\tilde{z}}_{0}$ be defined by solutions to the recurrence relation,

(20) $${\tilde{z}}_{0}^{i+2}=\left(\frac{\left(M-i)(\mu (0)+p(0)\right)}{\left(M-(i+1))p(0\right)}\right){\tilde{z}}_{0}^{i+1}-\left(\frac{\left(M-(i-1)\right)}{M-(i+1)}\right)\left(\frac{\mu (0)}{p(0)}\right){\tilde{z}}_{0}^{i},$$

for *i* = 1, 2, …, *M* − 2. It can be directly verified by induction that the solution to this recurrence relation is

(21) $${\tilde{z}}_{0}^{i}=\left(\frac{M}{M-(i-1)}\right){\left(\frac{\mu (0)}{p(0)}\right)}^{i-1}.$$

By construction of the recurrence relation, ${\left[\left(L_{U}-{\tilde{\lambda }}_{0}^{U}I\right){\tilde{z}}_{0}\right]}_{i}=0\;\text{for}\;i=2,3,\cdots ,M.$ The first component, ${\left[\left(L_{U}-{\tilde{\lambda }}_{0}^{U}I\right){\tilde{z}}_{0}\right]}_{1},$ satisfies

(22) $${\left[\left(L_{U}-{\tilde{\lambda }}_{0}^{U}I\right){\tilde{z}}_{0}\right]}_{1}=-\mu (0)M{\left(\frac{\mu (0)}{p(0)}\right)}^{M-1}\to 0$$

as *M* → ∞. Thus, when $M\gg 1,\left\|\left(L_{U}-{\tilde{\lambda }}_{0}^{U}I\right){\tilde{z}}_{0}\right\|\approx 0,$ and we may conclude that ${\tilde{\lambda }}_{0}^{U}=0\;\text{and}\;{\tilde{z}}_{0}$ are suitable approximations to $\lambda_{0}^{U}\;\text{and}\;z_{0},$ respectively. □

Recalling that all eigenvalues of *L*_*U*_ have negative real parts, the true eigenvalue $\lambda_{0}^{U}$ has a negative real part of very small magnitude. We now identify which entries in the eigenvector z_0_, as approximated by ${\tilde{z}}_{0},$ are particularly large in magnitude in comparison with the others. Recalling that the *i*-th component of *z*_0_ is given by ${\tilde{z}}_{0}^{i}=[M/(M-i+1)]{\left(\mu (0)/p(0)\right)}^{i-1},\;\text{the}\;{\tilde{z}}_{0}^{i}$ decay nearly exponentially in *i*, so that *c*_*k*_ for large *k* are preserved by this slow eigenvalue, in agreement with the described “build up” of clones at the boundary *k = M* when *γ* = 0.

The eigenvalue $\lambda_{1}^{U}$ of second smallest magnitude is well separated from $\lambda_{0}^{U},$ and it encodes information about how the number of small clones evolves. Similar analysis of an eigenvalue ${\tilde{\lambda }}_{1}^{U}$ and eigenvector ${\tilde{z}}_{1}$ approximating $\lambda_{1}^{U}\;\text{and}\;z_{1}$ indicates that *c*_*k*_ empties much more rapidly for small *k* than it does for large *k*, as small clones expand in size and race to the boundary at *k = M*. In particular, all *c*_*k*_ except those with *k ~ M*, which had been preserved by the slow eigenvalue $\lambda_{0}^{U},$ empty at nearly the same rate, $\lambda_{1}^{U}\approx {\tilde{\lambda }}_{1}^{U}=(\mu (0)-p(0)).$

#### Proposition 4.2.

*In the case p*(0) > *μ*(0), *the matrix L*_*U*_ *has an eigenvalue*, $\lambda_{1}^{U},$ *which is well approximated by* ${\tilde{\lambda }}_{1}^{U}=(\mu (0)-p(0)),$ *in the sense that there exists a vector* ${\tilde{z}}_{1}$ *such that* $\left\|\left(L_{U}-{\tilde{\lambda }}_{1}^{U}I\right){\tilde{z}}_{1}\right\|\to 0\;\text{as}\;M\to \infty .$

*Proof.* First define ${\tilde{z}}_{1}^{1}=1,\;\text{and}\;{\tilde{z}}_{1}^{2}=\left(\frac{M+1}{M-1}\right)\left(\frac{\mu (0)}{p(0)}\right)-\frac{1}{M-1}.$ Then for *i* = 1, 2, ... , *M* − 2, let ${\tilde{z}}_{1}^{i+1}$ be given by the solutions to the following recurrence relation:

(23) $${\tilde{z}}_{1}^{i+2}={\tilde{z}}_{1}^{i+1}+\left(\frac{M-(i-1)}{M-(i+1)}\right)\left(\frac{\mu (0)}{p(0)}\right)\left({\tilde{z}}_{1}^{i+1}-{\tilde{z}}_{1}^{i}\right).$$

It is worth nothing that if we were to instead choose ${\tilde{z}}_{1}^{1}={\tilde{z}}_{1}^{2},$ then the recurrence relation in Eq 23 would have a constant solution, ${\tilde{z}}_{1}^{i}={\tilde{z}}_{1}^{1}\;\text{for all}\;i=1,2,\cdots ,M.$ Although ${\tilde{z}}_{1}^{1}\neq {\tilde{z}}_{1}^{2}$ for our purposes, solutions of the recurrence relation do converge rapidly to constants, as will be discussed later.

By construction, ${\left[\left(L_{U}-{\tilde{\lambda }}_{1}^{U}I\right){\tilde{z}}_{1}\right]}_{i}=0\;\text{for}\;i=2,3,\cdots ,M+1.$ Additionally, ${\left[\left(L_{U}-{\tilde{\lambda }}_{1}^{U}I\right){\tilde{z}}_{1}\right]}_{1}=2\mu (0)\left({\tilde{z}}_{1}^{M}-{\tilde{z}}_{1}^{M-1}\right).$ To show that ${\left[\left(L_{U}-{\tilde{\lambda }}_{1}^{U}I\right){\tilde{z}}_{1}\right]}_{1}\to 0\;\text{as}\;M\to \infty ,$ we use Eq 23 to derive an explicit bound on the quantity $2\mu (0)\left({\tilde{z}}_{1}^{M}-{\tilde{z}}_{1}^{M-1}\right).$ Consider

(24) $$\left|{\tilde{z}}_{1}^{i+2}-{\tilde{z}}_{1}^{i+1}\right|=\left(\frac{M-(i-1)}{M-(i+1)}\right)\left(\frac{\mu (0)}{p(0)}\right)\left|{\tilde{z}}_{1}^{i+1}-{\tilde{z}}_{1}^{i}\right|\;\;=\left(\frac{M-(i-1)}{M-(i+1)}\right)\left(\frac{M-(i-2)}{M-i}\right){\left(\frac{\mu (0)}{p(0)}\right)}^{2}\left|{\tilde{z}}_{1}^{i}-{\tilde{z}}_{1}^{i-1}\right|\;\;\vdots \;\;=\left(\frac{M-(i-(j-1))}{M-(i+1)}\right)\left(\frac{M-(i-j)}{M-i}\right){\left(\frac{\mu (0)}{p(0)}\right)}^{j}\left|{\tilde{z}}_{1}^{i+2-j}-{\tilde{z}}_{1}^{i+1-j}\right|\;\;\vdots \;\;=\left(\frac{M-1}{M-(i+1)}\right)\left(\frac{M}{M-i}\right){\left(\frac{\mu (0)}{p(0)}\right)}^{i}\left|{\tilde{z}}_{1}^{2}-{\tilde{z}}_{1}^{1}\right|.$$

Taking *i* = *M* − 2 in the above relation, we find

$${\left\|\left(L_{U}-{\tilde{\lambda }}_{1}^{U}I\right){\tilde{z}}_{1}\right\|}_{1}=2\mu (0)\left|{\tilde{z}}_{1}^{M}-{\tilde{z}}_{1}^{M-1}\right|\;\;=\mu (0)(M(M-1)){\left(\frac{\mu (0)}{p(0)}\right)}^{M-2}\left|{\tilde{z}}_{1}^{2}-{\tilde{z}}_{1}^{1}\right|\to 0$$

as *M* → ∞. Thus, for *M* ≫ 1, $\left\|\left(L_{U}-{\tilde{\lambda }}_{1}^{U}I\right){\tilde{z}}_{1}\right\|\approx 0,$ and we find that ${\tilde{z}}_{1}$ is “almost” an eigenvector of *L*_*U*_ corresponding to the approximate eigenvalue ${\tilde{\lambda }}_{1}^{U}.$ □

We now wish to identify which of the *c*_*k*_ empty at the rate determined by the second approximate eigenvalue, ${\tilde{\lambda }}_{1}^{U}.$ As it turns out, the eigenvector ${\tilde{z}}_{1}$ corresponding to this eigenvalue is “nearly” constant, and thus all *c*_*k*_ empty at essentially the same rate. We see this by identifying that even though we are only concerned with a finite number (*M*) of terms of the sequence generated by the recurrence relation in Eq 23, as the index *M* becomes infinitely large, the sequence ${\left\{z_{1}^{i}\right\}}_{i=1}^{M}$ exhibits “Cauchy-like” behavior, mimicking “convergence” to a limiting value. We make this more precise in the following proposition:

#### Proposition 4.3.

*Let* ${\tilde{z}}_{1}$ *be an approximate eigenvector of L*_*U*_ *corresponding to approximate eigenvalue* ${\tilde{\lambda }}_{1}^{U},$ *where* ${\tilde{z}}_{1}$ *is generated by the recurrence relation in Eq 23. Then at large M, the components of* ${\tilde{z}}_{1}$ *exhibit “Cauchy-like” behavior: for any ε* > 0 *and* 0 < *c* < 1, *we may choose* *M* ∈ **N** *such that* $\left|{\tilde{z}}_{1}^{m}-{\tilde{z}}_{1}^{n}\right|<\varepsilon$ *for all cM* ≤ *m* ≤ *M and cM* ≤ *n* ≤ *M*.

*Proof.* Recalling the bound on $\left|{\tilde{z}}_{1}^{i+2}-{\tilde{z}}_{1}^{i+1}\right|$ obtained in Eq 24, we find

$$\left|{\tilde{z}}_{1}^{m}-{\tilde{z}}_{1}^{n}\right|=\left|\sum_{i=n}^{m-1}\left({\tilde{z}}_{1}^{i+1}-{\tilde{z}}_{1}^{i}\right)\right|\leq \sum_{i=n}^{m-1}\left|{\tilde{z}}_{1}^{i+1}-{\tilde{z}}_{1}^{i}\right|\;\;=\sum_{i=n}^{m-1}\left(\frac{M-1}{M-i}\right)\left(\frac{M}{M-(i-1)}\right){\left(\frac{\mu (0)}{p(0)}\right)}^{i-1}\left|{\tilde{z}}_{1}^{2}-{\tilde{z}}_{1}^{1}\right|\;\;\leq M(M-1)\left|{\tilde{z}}_{1}^{2}-{\tilde{z}}_{1}^{1}\right|\sum_{i=n}^{m-1}{\left(\frac{\mu (0)}{p(0)}\right)}^{i-1}\;\;=M(M-1)\left|{\tilde{z}}_{1}^{2}-{\tilde{z}}_{1}^{1}\right|{\left(\frac{\mu (0)}{p(0)}\right)}^{n-1}\left(\sum_{j=0}^{m-n-1}{\left(\frac{\mu (0)}{p(0)}\right)}^{j}\right)\;\;=M(M-1)\left|{\tilde{z}}_{1}^{2}-{\tilde{z}}_{1}^{1}\right|{\left(\frac{\mu (0)}{p(0)}\right)}^{n-1}\left(\frac{1-{\left(\frac{\mu (0)}{p(0)}\right)}^{m-n}}{1-\frac{\mu (0)}{p(0)}}\right)\;\;\leq \frac{\left|{\tilde{z}}_{1}^{2}-{\tilde{z}}_{1}^{1}\right|}{\left(1-\frac{\mu (0)}{p(0)}\right)}M(M-1){\left(\frac{\mu (0)}{p(0)}\right)}^{n-1}\leq \frac{\left|{\tilde{z}}_{1}^{2}-{\tilde{z}}_{1}^{1}\right|}{\left(1-\frac{\mu (0)}{p(0)}\right)}\left[M(M-1){\left(\frac{\mu (0)}{p(0)}\right)}^{cM-1}\right].$$

Recalling that $M(M-1){\left(\frac{\mu (0)}{p(0)}\right)}^{cM}\to 0\;\text{as}\;M\to \infty ,$ we may choose *M* large enough that

(25) $$M(M-1){\left(\frac{\mu (0)}{p(0)}\right)}^{cM}\leq \varepsilon \frac{\left[1-\left(\frac{\mu (0)}{p(0)}\right)\right]\left(\frac{\mu (0)}{p(0)}\right)}{\left|{\tilde{z}}_{1}^{2}-{\tilde{z}}_{1}^{1}\right|},$$

allowing us to conclude that $\left|{\tilde{z}}_{1}^{m}-{\tilde{z}}_{1}^{n}\right|<\varepsilon$ for all *M* ≥ *m, n* ≥ *cM*. □

By taking 0<c,ε≪1, we find that “most” components of ${\tilde{z}}_{1}$ are within an *ε*-distance of each other, so that the eigenvector is nearly constant. (The constancy breaks down at the components representing large-*k* compartments.) With this, we are able to classify the rates at which all of the *c*_*k*_ are lost from the pool. As the eigenvector corresponding to the second smallest magnitude eigenvalue, $\lambda_{1}^{U}\approx {\tilde{\lambda }}_{1}^{U},$ is nearly constant, all *c*_*k*_, except those with *k ~ M*, empty at nearly the same rate, on a time scale $\sim |\mu (0)-p(0){|}^{-1}.$

**Figure 4.**
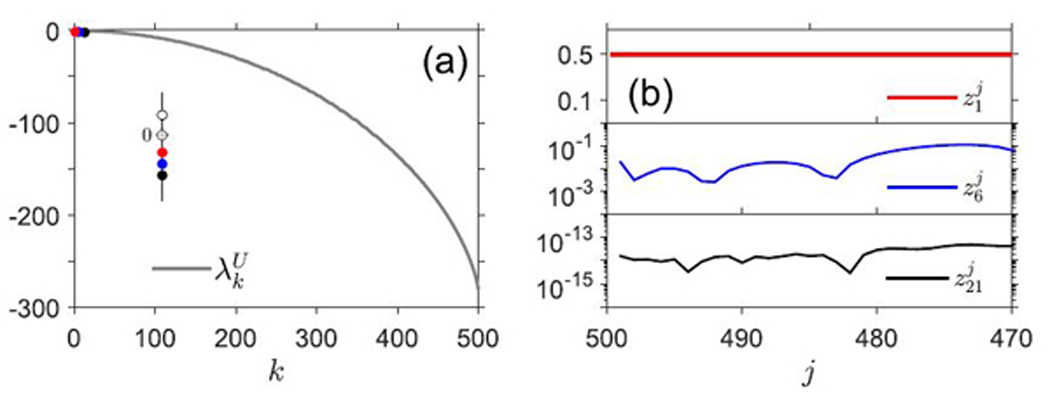
Eigenvalues and eigenvectors of $L_{\tilde{S}},\gamma =0.$ (a) Numerically computed eigenvalue spectrum of the matrix *L*_*U*_, with *p*(0) = 0.17, *μ*(0) = 0.12, and *M* = 500. Dots identify the locations of the eigenvalues $\lambda_{1}^{U}(\text{red}),\;\lambda_{6}^{U}\;(\text{blue}),\;\lambda_{21}^{U}\;(\text{black}).$ The first several eigenvalues of the full system, including the positive eigenvalue (open circle when *p*(0) > *μ*(0)) and the eigenvalue $\lambda_{0}^{U}\approx 0,$ are highlighted in the zoomed-in axis. (b) First 30 components $\left(z_{k}^{500},\cdots ,z_{k}^{470}\right)$ of the eigenvectors with indices *k* = 1, 6, 21, the eigenvalues corresponding to which are marked on the spectral curve in (a). Note that the eigenvectors were defined in a “reverse-order”, so that $z_{k}^{500}$ corresponds to compartment $c_{1},z_{k}^{495}$ to compartment *c*_6_, and $z_{k}^{480}$ to compartment *c*_21_. Generally, $z_{k}^{M-j+1}$ corresponds to compartment *c*_*j*_.

The remaining eigenvalues $\lambda_{2}^{U},\cdots ,\lambda_{M-1}^{U}$ are not treated analytically, but numerical computation indicates that a similar general approximation to the *k*-th eigenvalue, ${\tilde{\lambda }}_{k}^{U},$ may be made, taking the form ${\tilde{\lambda }}_{k}^{U}=k(\mu (0)-p(0)).$ The true eigenvalues, $\lambda_{k}^{U},$ are depicted in Figure 4a (comparison of the true and approximate spectra is omitted, as the result is similar to that depicted in Figure 1c. That is, $\lambda_{k}^{U}\approx k(\mu (0)-p(0))\;\text{if}\;k\leq M/10.).$ The oscillatory behavior observed in the approximate eigenvectors in the case *p*(N*) < *μ*(N*) of section 3 is absent here; although the subsequent approximate eigenvectors ${\tilde{z}}_{2},\cdots ,{\tilde{z}}_{M-1}$ do not share the Cauchy-like behavior of ${\tilde{z}}_{1},$ the components corresponding to *c*_*k*_ for small *k* do not vary much in magnitude, and are thus interpreted as being nearly constant themselves (Figure 4b). Thus, we conclude that for small *k*, the functions *c*_*k*_ all have very similar dynamics, diverging at the rate |μ(0)−p(0)|, while for large *k*, the *c*_*k*_ converge very slowly, at a rate governed by the dominant, near-zero eigenvalue $\lambda_{0}^{U}.$

We now consider the stable equilibrium solution, $c_{k\leq M}^{*}=0\;\text{for}\;k\geq 1,N^{*}=\tilde{N}>0.$ In this case, the linearization around this equilibrium may be expressed as $p\left(N^{*}\right)L_{U^{\prime }},\;\text{where}\;L_{U^{\prime }}={\left(u_{ij}^{\prime }\right)}_{1\leq i,j\leq M+1}\;\text{is the}\;(M+1)\times (M+1)$ matrix with component $u_{ij}^{\prime }$ given by

(26) $$\left\{\begin{array}{ll} -2j, & \text{if}\;i=j\leq M-1\\ -M, & \text{if}\;i=j=M\\ j, & \text{if}\;i=j-1;2\leq j\leq M\\ j, & \text{if}\;i=j+1;1\leq j\leq M-1\\ \frac{p^{\prime }\left(N^{*}\right)N^{*}+p\left(N^{*}\right)-\mu^{\prime }\left(N^{*}\right)N^{*}-\mu \left(N^{*}\right)}{p\left(N^{*}\right)}, & \text{if}\;i=j=M+1\\ 0. & \text{otherwise} \end{array}\right\}$$

For reference, an example of the matrix $L_{U^{\prime }},$ with *M* = 4, is given below:

(27) $$\left(\begin{array}{ccccc} -2 & 2 & 0 & 0 & 0\\ 1 & -4 & 3 & 0 & 0\\ 0 & 2 & -6 & 4 & 0\\ 0 & 0 & 3 & -4 & 0\\ 0 & 0 & 0 & 0 & \frac{p^{\prime }\left(N^{*}\right)N^{*}+p\left(N^{*}\right)-\mu^{\prime }\left(N^{*}\right)N^{*}-\mu \left(N^{*}\right)}{p\left(N^{*}\right)} \end{array}\right)$$

Denote by $\lambda_{k}^{U^{\prime }}\;\text{for}\;k=1,2,\cdots ,M,M+1$ the eigenvalues of the matrix $L_{U^{\prime }}$ evaluated at the stable equilibrium solution, and $x_{k}=\left(x_{k}^{1},x_{k}^{2},\cdots ,x_{k}^{M},x_{k}^{M+1}\right)$ their corresponding eigenvectors. As before, let $\lambda_{M+1}^{U^{\prime }}={\frac{1}{p\left(N^{*}\right)}\frac{\partial [(p-\mu )N]}{\partial N}|}_{N=N^{*}}<0.$ Then, the remaining eigenvalues $\lambda_{1}^{U^{\prime }},\cdots ,\lambda_{M}^{U^{\prime }}$ are those of the $(M+1)\times (M+1)\;\text{minor of}\;L_{U^{\prime }},$ which are independent of the parameters of the system except *M*. (Of course, the eigenvalues of $p\left(N^{*}\right)L_{U^{\prime }}\;\text{are then}\;p\left(N^{*}\right)\lambda_{k}^{U^{\prime }}\;\text{for}\;k=1,\cdots ,M+1$.) These eigenvalues and eigenvectors are not treated analytically. The analysis conducted in section 3 does not apply, as it relied on the assumption *p*(N*) < *μ*(N*), which no longer holds. However, numerical computation indicates that in the case *p*(N*) = *μ*(N*), which applies here, the eigenvalue spectrum and associated eigenvectors qualitatively resemble the $\lambda_{k}^{S},y_{k}$ studied analytically in section 3.

### 4.3 Behavior of the linearized and fully nonlinear systems

We now interpret these results in the context of the particular diseased states to which they naturally apply. We first identified an unstable equilibrium solution, $c_{k}^{*}=0,N^{*}=0,$ and studied the linearization of the system around this equilibrium. Under the linearized model, if *γ*_*i*_ = 0 for some *i*, the eigenvalue/eigenvectors pairs suggest that solutions diverge away from this equilibrium, with $c_{k}^{i}$ for small *k* evolving at a rate $\sim \lambda_{1}^{U}=(\mu (0)-p(0)),\;\text{and}\;c_{k}^{i}$ for *k* − *M* evolving at the very slow rate given by the small-magnitude eigenvalue, $\lambda_{0}^{U}.$ The total population *N*^*i*^(*t*) evolves at the rate (*p*(0) − *μ*(0)). This situation represents the repopulation of the T cell pool from a small number of cells via peripheral proliferation in a highly pathological state involving both complete thymic inactivity (e.g. thymectomy or total functional cessation) and near full lymphopenia (as may result from treatment regimens for cancer, etc.).

We then identified a stable equilibrium solution, $c_{k}^{*}=0,N^{*}=\tilde{N}>0.\;\text{As}\;\tilde{N}$ is asymptotically stable, $N^{i}(t)\to \tilde{N}$ after diverging from $N^{*}=0.$ Under the linearized model, the eigenvalue/eigenvector pairs suggest that $c_{k}^{i}(t)\to 0$ slowly for small *k*, and $c_{k}^{i}(t)\to 0$ much more quickly for large *k*.

As before, the validity of the eigenvalues in providing accurate convergence rates of $c_{k}^{i},N^{i}$ to and from equilibria depends on the initial condition $c_{k}^{i}\left(t_{i}\right)$ in the full nonlinear model. If the human is in a state of immune health for $t<t_{i},$ so that *γ*_*i*−1_ > 0 and the initial conditions $c_{k}^{i}\left(t_{i}\right),N^{i}\left(t_{i}\right)>0$ satisfy $c_{k}^{i}\left(t_{i}\right)\sim c_{k}^{*}\left(\gamma_{i-1}\right),N^{i}\left(t_{i}\right)\sim N^{*}\left(\gamma_{i-1}\right),$ we expect that $p\left(N^{i}\left(t_{i}\right)\right)<\mu \left(N^{i}\left(t_{i}\right)\right).$ The higher rate of death than proliferation at *t*_*i*_ may cause a transient period of quick collapse, with *N*^*i*^(*t*) decreasing to $\tilde{N}.\;\text{As}\;N^{i}(t)\to \tilde{N},$ convergence occurs at the rates dictated by the linearized eigenvalues. If *γ*_*i*−1_ > 0 but $c_{k}^{i}\left(t_{i}\right),N^{i}\left(t_{i}\right)\sim 0,$ so that the thymus is functioning to some extent but the T cell pool has been eradicated, trajectories first diverge away from the unstable zero equilibrium at rates given by the linearized eigenvalues. As $p\left(N^{i}\left(t_{i}\right)\right)-\mu \left(N^{i}\left(t_{i}\right)\right)\to p(\tilde{N})-\mu (\tilde{N})=0,$ the motion of trajectories transitions from being dictated by the eigenvalues of the unstable equilibrium to those of the stable equilibrium.

In summary, we show in Proposition 4.1 that *c*_*k*_ for larger *k* is sustained by a near-zero eigenvalue $\lambda_{0}^{U}≃0,$ as the solution evolves away from the unstable empty state when *γ* = 0. Furthermore, in Propositions 4.2 and 4.3 we identify a series of negative eigenvalues, and a uniform eigenvector corresponding to the slowest decay rate $\lambda_{1}^{U}<0.$ It suggests a uniform asymptotic decay of components other than the larger *k* components preserved by $\lambda_{0}^{U}.$

## 5. Special cases and numerical evaluation

Using the approximate rates of convergence provided by linearization, we can now study the time scale of the T cell pool’s adjustment to a new export rate. While some T cell clones will expand and attain a large size, most are small. Thus, in both the cases of thymic atrophy and recovery, we take as a proxy for the rate at which the T cell diversity converges to equilibrium the eigenvalue that dictates the rate of convergence of *c*_1_, typically given by the quantity *p*(N*) − *μ*(N*) (this tends to also be the dominant eigenvalue). Recalling that *p*(N*) < *μ*(N*), high proliferation rates (*p*(N*) ~ *μ*(N*)) lead to small values of |*p*(N*) – *μ*(N*)|, thus slower adjustment of diversity to the changing *γ*. That is, in a proliferation-dominant scenario, a drop in thymic export leads to repopulation via clonal expansion. In this section, we study several specific models arising from canonical choices of *p* and *μ*, and compute the changing convergence rate as gamma varies.

### 5.1 The logistic mode: Regulated proliferation, constant death

We begin with the canonical logistic growth model, taking *p*(*N*) = *p*_0_(1 − *N*/*K*), *μ*(*N*) = *μ*_o_, where *p*_0_, *μ*_0_ > 0 are basal rates of cellular proliferation and death, respectively, and *K* > 0 is an inherent carrying capacity. Under this model, Eq 1 has a positive steady state, N*, given by

(28) $$N^{*}=\left(\frac{K}{2p_{0}}\right)\left(\left(p_{0}-\mu_{0}\right)+\sqrt{{\left(p_{0}-\mu_{0}\right)}^{2}+\frac{4\gamma p_{0}}{K}}\right).$$

In this case, $p\left(N^{*}\right)-\mu \left(N^{*}\right)=p_{0}\left(1-\frac{N^{*}}{K}\right)-\mu_{0}=\frac{1}{2}\left(\left(p_{0}-\mu_{0}\right)-\sqrt{{\left(p_{0}-\mu_{0}\right)}^{2}+\frac{4\gamma p_{0}}{K}}\right)<0,$ so that the assumption $p\left(N^{*}\right)<\mu \left(N^{*}\right)$ always applies. Moreover, ${\tilde{\lambda }}_{M+1}^{S}=-\sqrt{{\left(p_{0}-\mu_{0}\right)}^{2}+\frac{4\gamma p_{0}}{K}},$ so it is clear that $0>{\tilde{\lambda }}_{1}^{S}>{\tilde{\lambda }}_{M+1}^{S},\;\text{and}\;{\tilde{\lambda }}_{1}^{S}$ is the dominant eigenvalue. Then,

(29) $$\left|p\left(N^{*}\right)-\mu \left(N^{*}\right)\right|=\frac{1}{2}\left(-\left(p_{0}-\mu_{0}\right)+\sqrt{{\left(p_{0}-\mu_{0}\right)}^{2}+\frac{4\gamma p_{0}}{K}}\right).$$

**Figure 5.**
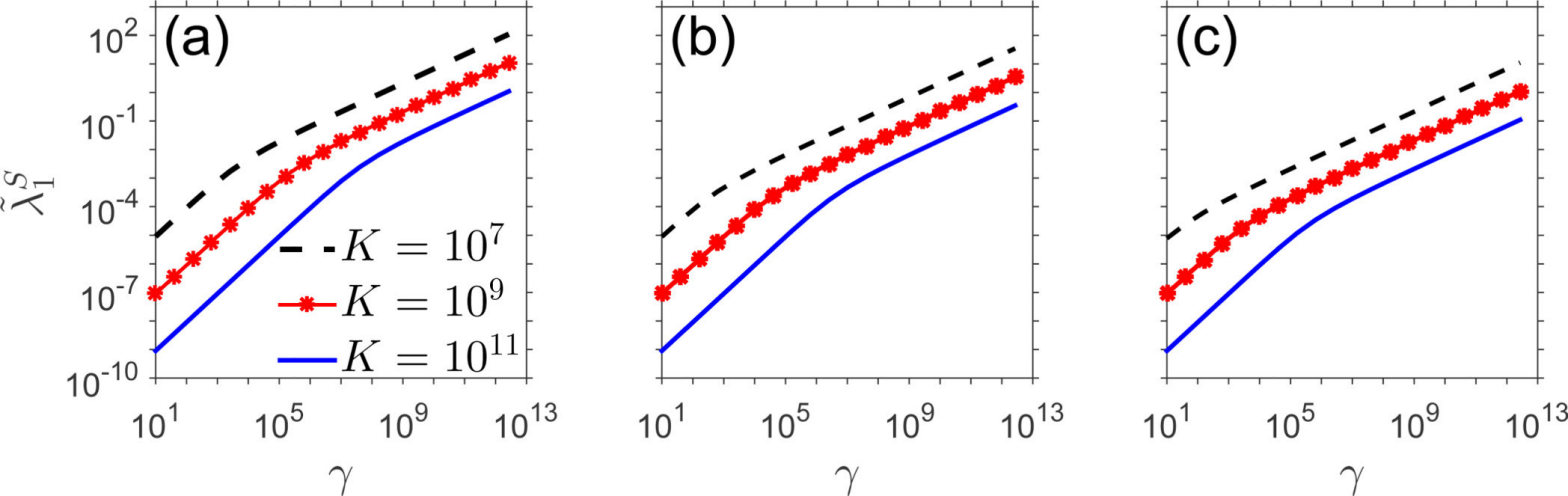
Dominant eigenvalue, ${\tilde{\lambda }}_{1}^{S},\;\text{of}\;L_{\tilde{S}},$ plotted against *γ*, case 1. In (a), *p*_0_ = 0.18, *μ*_0_ = 0.17. In (b), *p*_0_ = 0.018, *μ*_0_ = 0.017. In (c), *μ*_0_ = 0.0018, *μ*_0_ = 0.0017. There is an approximate power law relationship between ${\tilde{\lambda }}_{1}^{S}\;\text{and}\;\gamma$ within this range of parameter values. In (a), for example, the best fit line to the curve *K* = 10^7^ is given by $\log{\tilde{\lambda }}_{1}^{S}=0.5731\;\log\gamma -4.869,$ The curve *K* = 10^9^ is fit by $\log{\tilde{\lambda }}_{1}^{S}=0.6749\;\log\gamma -6.894,$ with *R*^*2*^ = 0.9752, and the curve *K* = 10^11^ is fit by $\log{\tilde{\lambda }}_{1}^{S}=0.7939\;\log\gamma -9.206,$ with *R*^2^ = 0.9801.

In Figure 5, the quantity ${\tilde{\lambda }}_{1}^{S}$ is plotted against *γ* for several different combinations of *p*_0_,*μ*_0_, showing the unboundedness of the convergence rate as *γ* increases. Within this physiological range of *γ* values, the dependence of ${\tilde{\lambda }}_{1}^{S}$ on *γ* presents as linear on a log-log plot, indicating a power law relationship. Indeed, the power law is described in detail in the caption of Figure 5.

### 5.2 Constant proliferation, regulated death

Let us now assume that $p(N)=p_{0}>0\;\text{and}\;\mu (N)=\mu_{0}+\frac{\mu_{1}N^{2}}{K^{2}+N^{2}},$ with *μ*_0_, *μ*_1_ > 0, in the determination of *N*(*t*) via Eq 1. We assume that $p_{0}>\mu_{0}\;\text{and}\;p_{0}-\left(\mu_{0}+\mu_{1}\right)<0,$ so that the action of the proliferation-death mechanism results in net cellular birth at low cell counts and net cellular death at high cell counts. The steady states of Eq 1 are given by the roots of the following cubic

(30) $$P(N)=\left(p_{0}-\left(\mu_{0}+\mu_{1}\right)\right)N^{3}+\gamma N^{2}+\left(p_{0}-\mu_{0}\right)K^{2}N+\gamma K^{2}.$$

First note that *P*(0) = *γK*^2^ > 0, and the highest order coefficient satisfies (*p*_0_ − (*μ*_0_ + *μ*_1_)) < 0 by assumption, so that *P*(*N*) → −∞ as *N* → ∞, and *P*(*N*) has at least one positive, real root. From Descartes’ rules of signs, the polynomial has at most one positive real root, so we may conclude that it has precisely one positive real root. This root corresponds to the only physically relevant stable fixed point of $\frac{\text{d}N}{\text{d}t}.$ By regarding this root, N*, as the intersection of the line $\gamma +\left(p_{0}-\mu_{0}\right)N$ and the rational expression $\mu_{1}\left(\frac{N^{3}}{K^{2}+N^{2}}\right),$ we see that N* → ∞ as *γ* → ∞.

We also verify that the eigenvalues ${\tilde{\lambda }}_{1}^{S},{\tilde{\lambda }}_{M+1}^{S}$ satisfy $0>{\tilde{\lambda }}_{1}^{S}>{\tilde{\lambda }}_{M+1}^{S},\;\text{so that}\;{\tilde{\lambda }}_{1}^{S}$ is, in fact, the dominant eigenvalue. We first check that $0\;>\;{\tilde{\lambda }}_{1}^{S}.$ Recalling that ${\tilde{\lambda }}_{1}^{S}=p\left(N^{*}\right)-\mu \left(N^{*}\right)=p_{0}-\left(\mu_{0}+\mu_{1}\left(\frac{{\left(N^{*}\right)}^{2}}{\left(K^{2}+{\left(N^{*}\right)}^{2}\right)}\right)\right),$ we see after some simple algebraic manipulation that the condition ${\tilde{\lambda }}_{1}^{S}<0$ is equivalent to

(31) $$N^{*}>{\left(\frac{\left(p_{0}-\mu_{0}\right)K^{2}}{\left|p_{0}-\left(\mu_{0}+\mu_{1}\right)\right|}\right)}^{\frac{1}{2}}:=\bar{N}.$$

But $P(\bar{N})=\frac{\gamma \left(p_{0}-\mu_{0}\right)K^{2}}{\left|p_{0}-\left(\mu_{0}+\mu_{1}\right)\right|}+\gamma K^{2}>0.\;\text{That}\;P(\bar{N})>0\;\text{and}\;P(N)\to -\infty \;\text{as}\;N\to \infty ,$ to, along with the fact that *P*(*N*) has only one real positive root indicates that N* does, in fact, satisfy Eq 31, so that ${\tilde{\lambda }}_{1}^{S}<0.$ It is easily verified that $-\mu^{\prime }\left(N^{*}\right)N^{*}<0,$ and consequently that ${\tilde{\lambda }}_{1}^{S}>{\tilde{\lambda }}_{M+1}^{S}.$

**Figure 6.**
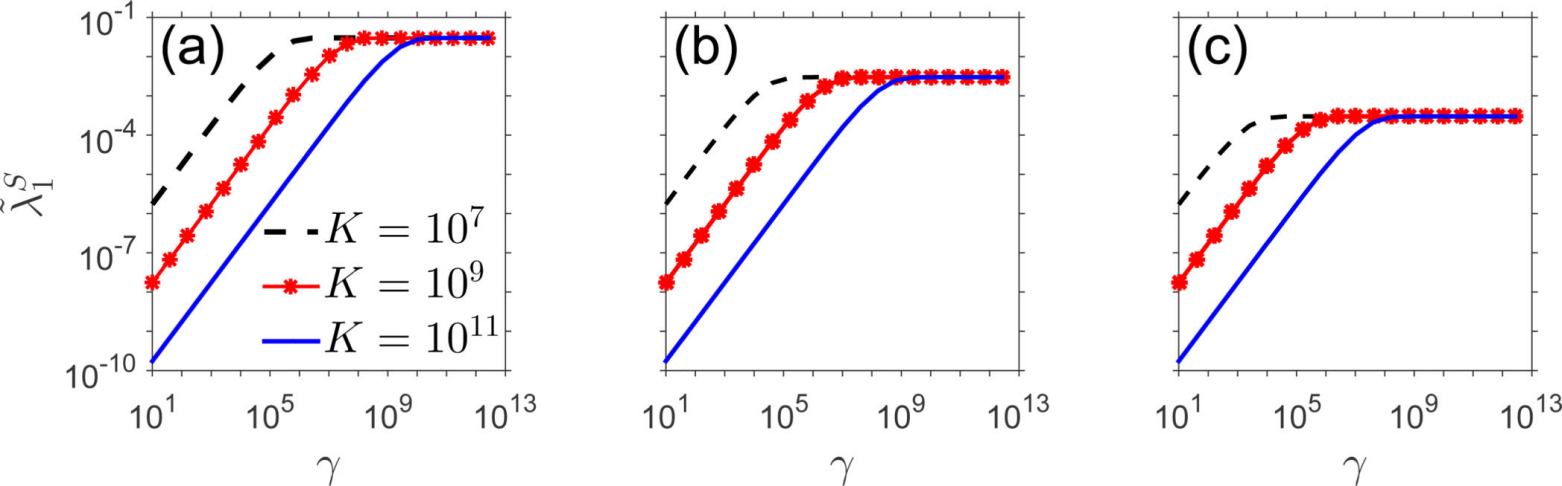
Dominant eigenvalue, ${\tilde{\lambda }}_{1}^{S},\;\text{of}\;L_{\tilde{S}},$ plotted against *γ* case 2. In (a), *p*_0_ = 0.18, *μ*_0_ = 0.17, and *μ*_1_ = 0.004. In (b), *p*_0_ = 0.018, *μ*_0_ = 0.017, and *μ*_1_ = 0.004. In (c), *p*_0_ = 0.0018, *μ*_0_ = 0.0017, and *μ*_1_ = 0.0004. The relationship between ${\tilde{\lambda }}_{1}^{S}\;\text{and}\;\gamma$ follows a power law for low values of *γ*, before reaching a plateau for high values of *γ*. In (a), the best fit line to the curve *K* = 10^7^ over the power law region $\left(\sim \gamma \in \left[10^{1},10^{5}\right]\right)$ is given by $\log{\tilde{\lambda }}_{1}^{S}=0.9606\;\log\gamma -6.679,$ with *R*^2^ = 0.9989. The curve *K* = 10^9^ over the power law region $\left(\sim \gamma \in \left[10^{1},10^{7}\right]\right)$ is fit by ${\tilde{\lambda }}_{1}^{S}=0.9807$ log *γ* − 8.708, with *R*^2^ = 0.9995, and the curve *K* = 10^11^ over the power law region $\left(\sim \gamma \in \left[10^{1},10^{9}\right]\right)$ is fit by $\log{\tilde{\lambda }}_{1}^{S}=0.9883$ log *γ* − 10.72, with *R*^2^ = 0.9998.

From the fact that N* → ∞ as *γ* → ∞, we have that |*p*(N*) − *μ*(N*)| → *p*_0_ − (*μ*_0_ + *μ*_1_) as *γ* → ∞. This limiting behavior is reflected in the eventual plateau seen in Figure 6, which plots the quantity ${\tilde{\lambda }}_{1}^{S}$ in this case. Before the plateau occurs, *γ* and ${\tilde{\lambda }}_{1}^{S}$ are again related by a power law. The transition from power law to plateau occurs at a “threshold” value, γ*, of *γ*, at which the rate of T cell adjustment becomes sensitive to a changing thymic export rate. If, for some $i,\gamma_{i-1},\gamma_{i}\geq \gamma^{*},{\tilde{\lambda }}_{1}^{S}$ is unaffected by the transition from thymic export rate *γ*_*i*−1_ to thymic export rate *γ*_*i*_-that is, the T cell pool adjusts to the new thymic export rate *γ*_*i*_ as quickly as it had adjusted to the previous thymic export rate *γ*_*i*−1_. If, however, $\left(\gamma_{i}-\gamma^{*}\right)\left(\gamma_{i-1}-\gamma^{*}\right)<0,$ then a dramatic shift in the adjustment rate will occur. Thus, parameter choices that result in a low threshold value γ* might correspond to physiological conditions under which an instance of acute thymic atrophy actually does not affect T cell adjustment rates. Likewise, a high threshold value of γ* indicates potential sensitivity of adjustment rates to the changing level of thymic export, with adjustment rates obeying a power law dependence on *γ*.

## 6. Discussion and conclusions

In this paper, we formulated a model of how the naive T cell pool adjusts to changes in the rate of thymic export of new T cells during a cycle of stress-induced atrophy and recovery, and how it may be reconstituted following an instance of severe lymphopenia induced by a state of immune disease, or treatments such as chemotherapy. Our underlying model is a birth-death-immigration process studied under a mean-field approximation for the mean clone abundance distribution (or clone count) *c*_*k*_(*t*). A recent investigation into the fully stochastic model indicates that the true *c*_*k*_ differs from the *c*_*k*_ derived using the mean-field assumption (Eqs 2–5) only for very large k≈N [52]. Thus, our analyses may be inaccurate only if a single large clone dominates the whole population.

Another modeling choice we made is that TCRs are generated one naive T cell at a time. Successive emigrations from the thymus are uncorrelated with the TCRs that are produced. However, emigration can be clustered, with cell proliferation generating Δ ~ 2 − 4 copies of naive T cells carrying the same TCR during each emigration event. In this case, we simply modify the immigration terms in Eqs 2–3. For Eq 2, the immigration term proportional to *γ*/Ω is removed, while the immigration term $\gamma /\Omega \left(c_{k-1}-c_{k}\right)$ is replaced by $\gamma /\Omega \left(c_{k-\Delta }-c_{k}\right)$ in Eq 3. By setting $c_{l<0}=0,$ the solution to Eqs 2 and 3 can be numerically evaluated but a closed-form analytic solution is not possible. Solutions with clustered immigration (Δ > 1) show no qualitative difference from Δ = 1, with minor quantitative differences arising only for very small *k*.

In section 3, we found that our mean-field ODE model admitted one stable equilibrium solution when *γ* > 0. From an analysis of the eigenvalues and eigenvectors of the system linearized around this stable equilibrium, we found that for small *k*, perturbations in *c*_*k*_ about a steady-state solution are weighted more strongly in the slowest mode (slowest eigenvalue) $\lambda_{1}^{S}=p\left(N^{*}\right)-\mu \left(N^{*}\right)<0.$ As also shown in Figure 1, the variation in *c*_*k*_ for larger *k* contains higher weights of the faster modes corresponding to more negative (faster) eigenvalues $\lambda_{l}^{S}=l\left(p\left(N^{*}\right)-\mu \left(N^{*}\right)\right)<0.$ Similarly, in section 4, we analyzed the eigenvalue and eigenvector decomposition of the solution for *γ* = 0, for which two equilibrium points, N* = 0 and N* > 0, arise. For *p*(N*) − *μ*(N*) < 0, we find the same decomposition of *c*_*k*_(*t*) as in the *γ* > 0 case in section 3. Thus, in terms of the relaxation of *c*_*k*_(*t*) towards a finite steady-state, our eigenvalue/eigenvector analysis suggests that the counts of large clones might evolve faster towards the new steady-state. For the unstable equilibrium state N* = 0, the eigenvalue/eigenvector decomposition of *c*_*k*_ is shown in Figure 4. In this unstable case, the slowest eigenvalue $\lambda_{0}^{U}\approx 0$ has a corresponding eigenvector $z_{0}^{i}$ with elements that decay with *i*. This result predicts that large-population (*M* − *i*) clones relax slowly (recall that the labeling is inverted: *i* = *M* − 1 corresponds to *c*_1_).

In addition to decomposing the linearized solutions in terms of eigenvalues and eigenvectors, we explicitly plot trajectories of *c*_*k*_(*t*) following small, abrupt changes in *γ*.

**Figure 7.**
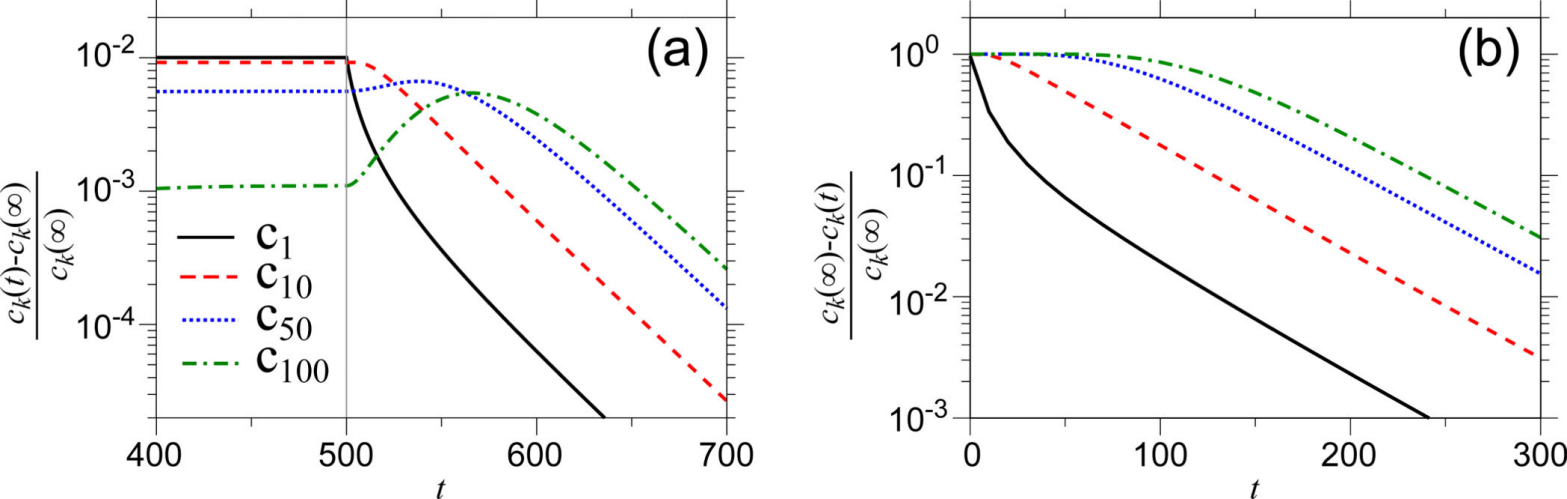
Time-dependence of *c*_*k*_(*t*) near fixed points. (a) The explicit time-evolution of $\delta c_{k}=\left(c_{k}(t)-c_{k}(\infty )\right)/c_{k}(\infty )$ following a small abrupt change *γ* = 1.8 × 10^10^ → 0.99 × 1.8 × 10^10^ at *t* = 0. The evolution from one nonzero steady state to another nonzero steady state shows that clone counts of small clones appear to evolve faster than counts of larger clones. (b) Similarly, the number of small clones also evolve faster away from an unstable empty equilibrium state N* = 0.

By plotting the log of the deviation $\delta c_{k}=\left(c_{k}(t)-c_{k}(\infty )\right)/c_{k}(\infty )$ in *all* cases (Figure 7a), we can see that counts of *small* clones evolve faster after perturbation. Although this seems to contradict the eigen decomposition of the *γ* > 0 case, the coefficients and eigenvector elements corresponding to larger l are negative (see Figure 1b,d), and convert fast decaying modes into short bursts of growth and conspire to cancel the fast dynamics indicated by the more negative eigenvalues. In fact, we see that the clone counts of large clones actually evolve more slowly than the rate associated with the largest eigenvalue. This behavior can also be heuristically understood by noticing that under a given change in *γ*, the immigration term $\gamma \left(c_{k-1}-c_{k}\right)/\Omega$ is larger for smaller *k* because *c*_*k*_ ~ 1/*k*. Thus, for a given change in *γ*, the perturbation is larger in the equations with smaller *k* and induces changes in *c*_*k*_ (*t*) that appear larger.

In section 5, we infer the rate of convergence of the counts of the smallest (but most common) clones to equilibrium by computing the dominant eigenvalue as a function of *γ* > 0 for two choices of regulated functions *p*(*N*),*μ*(*N*). In section 5.1, we test the logistic model, which assumes a constant death rate but a population-dependent proliferation rate. From the explicit form of ${\tilde{\lambda }}_{1}^{S}$ in Eq 29, $p\left(N^{*}\right)-\mu \left(N^{*}\right)\to \infty \;\text{as}\;\gamma \to \infty$ for fixed values of the other parameters, which produces the power-law relationship between *γ* and ${\tilde{\lambda }}_{1}^{S}$ depicted in Figure 5. In section 5.2, we assumed instead a constant rate of cellular proliferation with an *N*-dependent death rate. In this case, which differs from the logistic formulation in that regulation is incorporated into *μ*(*N*) via a Hill-type function, *γ* and ${\tilde{\lambda }}_{1}^{S}$ are related by a power law for low *γ*, before reaching a plateau at higher *γ*.

Since regulation through death is typically associated with the actual mechanism of naive T cell survival, we expect this mechanism to be more realistic than a population-dependent proliferation rate *p*(*N*). Thus, jumps in the thymic export rate that either cross the threshold value of *γ*, or occur between two values of *γ* both in the power-law region, can be expected to produce changes in both the equilibrium values of *c*_*k*_ and also the convergence rates. If a jump in *γ* occurs between two values of *γ* that are both in the plateau region, the equilibrium values shift, but the convergence rates stay the same. The presence of the power law region indicates the robustness of the T cell diversity during a time of severe thymic atrophy. That is, the slower convergence of the T cell diversity to equilibrium at low *γ* values protects the pool from quick shifts to the lower diversity associated with lower *γ* values.

## Appendix A. Verification of recurrence relation solution

Here, we verify that Eq 16 satisfies the recurrence relation in Eq 15. For notational simplicity, we assume $p/\mu \equiv p\left(N^{*}\right)/\mu \left(N^{*}\right)$ is evaluated at the relevant steady state defined by *N**. Inserting Eq 16 into the right-hand-side of Eq 15, we find

$$\left[\left(i+(k-1))\left(\frac{p}{\mu }\right)+(i-(k+1)\right)\right]y_{k}^{i-1}-[i-2]\left(\frac{p}{\mu }\right)y_{k}^{i-2}$$

$$=\left[\frac{\left(i+(k-1)\right)}{i}\left(\frac{p}{\mu }\right)+\frac{i-(k+1)}{i}\right]\left[\frac{{\left(\frac{p}{\mu }\right)}^{i-2}}{k!}\prod_{j=1}^{k-1}\left(i-1+j\right)+\sum_{n=2}^{k-1}\left[\prod_{m=1}^{n-1}\left(i-1-m\right)\right]\left[\prod_{j=1}^{k-n}\left(i-1+j\right)\right]\frac{{\left(\frac{p}{\mu }\right)}^{i-1-n}}{{\left(-1\right)}^{n-1}(n-1)!k(k-n)!}+\frac{{\left(\frac{p}{\mu }\right)}^{i-1-k}}{{\left(-1\right)}^{k-1}k!}\prod_{j=1}^{k-1}\left(i-1-j\right)\right]$$

$$-\left(\frac{p}{\mu }\right)\left(\frac{i-2}{i}\right)\left[\left(\prod_{j=1}^{k-1}\left(i-2-j\right)\right)\frac{\left(\frac{p}{\mu }\right)}{k!}+\sum_{n=2}^{k-1}\left[\prod_{m=1}^{n-1}\left(i-2-m\right)\right]\left[\prod_{j=1}^{k-n}\left(i-2+j\right)\right]\frac{\left(\frac{p}{\mu }\right)}{{\left(-1\right)}^{n-1}(n-1)!k(k-n)!}+\frac{{\left(\frac{p}{\mu }\right)}^{i-2-k}}{{\left(-1\right)}^{k-1}k!}\prod_{j=1}^{k-1}\left(i-2-j\right)\right]$$

$$=\left[\prod_{j=0}^{k-1}\left(i+j\right)\right]\frac{{\left(\frac{p}{\mu }\right)}^{i-1}}{k!}+[\frac{\left(i-(k+1)\right)}{k!}\left[\prod_{j=1}^{k-1}\left(i-1+j\right)\right]-\frac{\left(i+(k-1)\right)}{k(k-2)!}\left[\prod_{j=1}^{k-2}\left(i-1+j\right)\right]-\frac{\left(i-2\right)}{k!}\left[\prod_{j=1}^{k-1}\left(i-2+j\right)\right]]{\left(\frac{p}{\mu }\right)}^{i-2}$$

$$+\sum_{n=2}^{k-1}(i-(k+1))\left[\prod_{j=1}^{n-1}(i-1-j)\right]\left[\prod_{j=1}^{k-n}\left(i-1+j\right)\right]\frac{{\left(\frac{p}{\mu }\right)}^{i-1-n}}{{\left(-1\right)}^{n-1}(n-1)!k(k-n)!}+\sum_{n=3}^{k-1}(i+(k-1))\left[\prod_{j=1}^{n-1}\left(i-1-j\right)\right]\left[\prod_{j=1}^{k-n}\left(i-1+j\right)\right]\frac{{\left(\frac{p}{\mu }\right)}^{i-n}}{{\left(-1\right)}^{n-1}(n-1)!k(k-n)!}$$

$$+(i+(k-1))\left[\prod_{j=1}^{k-1}\left(i-1-j\right)\right]\left[\frac{{\left(\frac{p}{\mu }\right)}^{i-k}}{{\left(-1\right)}^{k-1}k!}\right]+(i-(k+1))\left[\prod_{j=1}^{k-1}\left(i-1-j\right)\right]\frac{{\left(\frac{p}{\mu }\right)}^{i-1-k}}{{\left(-1\right)}^{k-1}k!}$$

$$-\sum_{n=2}^{k-1}\left(i-2\right)\left[\prod_{m=1}^{n-1}\left(i-2-m\right)\right]\left[\prod_{j=1}^{k-n}\left(i-2+j\right)\right]\frac{{\left(\frac{p}{\mu }\right)}^{i-1-n}}{{\left(-1\right)}^{n-1}(n-1)!k(k-n)!}-(i-2)\left[\prod_{j=1}^{k-1}\left(i-2-j\right)\right]\frac{{\left(\frac{p}{\mu }\right)}^{i-1-k}}{{\left(-1\right)}^{k-1}k!}$$

$$=\frac{{\left(\frac{p}{\mu }\right)}^{i-1}}{k!}\prod_{j=0}^{k-1}\left(i+j\right)+{\left(\frac{p}{\mu }\right)}^{i-2}\left[\frac{\left(i+(k-2))(i-(k+1))-(k-1)(i-2)(i+(k-1))-(i-2)(i-1\right)}{k!}\right]\prod_{j=0}^{k-3}\left(i+j\right)$$

$$+\sum_{s=3}^{k-1}\left([\prod_{j=1}^{s-1}\left(i-1-j\right)\right]\left[\prod_{j=1}^{k-s}(i-1+j)\right]\frac{\left(i+(k-1)\right)}{{\left(-1\right)}^{s-1}(s-1)!k(k-s)!}+\left[\prod_{j=1}^{s-2}(i-1-j)\right]\left[\prod_{j=1}^{k-(s-1)}\left(i-1+j\right)\right]\frac{\left(i-(k+1)\right)}{{\left(-1\right)}^{s-2}(s-2)!k(k-(s-1))!}-(i-2)\left[\prod_{j=1}^{s-2}(i-2-j)\right]\left[\prod_{j=1}^{k-(s-1)}\left(i-2+j\right)\right]\frac{1}{{\left(-1\right)}^{s-2}(s-2)!k(k-(s-1))!}){\left(\frac{p}{\mu }\right)}^{i-s}$$

$$+((i-(k+1))\left[\prod_{j=1}^{k-2}\left(i-1-j\right)\right]\frac{i}{{\left(-1\right)}^{k-2}(k-2)!k}+(i+(k-1))[\prod_{j=1}^{k-1}(i-1-j)]\frac{1}{k!}-(i-2)\left[\prod_{j=1}^{k-2}\left(i-2-j\right)\right]\frac{\left(i-1\right)}{{\left(-1\right)}^{k-2}(k-2)!k}){\left(\frac{p}{\mu }\right)}^{i-k}$$

$$=\left[\prod_{j=0}^{k-1}\left(i+j\right)\right]\frac{{\left(\frac{p}{\mu }\right)}^{i-1}}{k!}+\left[\prod_{j=0}^{k-3}\left(i+j\right)\right]\left[\frac{-(k-1)i^{2}-(k-1)(k-3)i+(k-2)(k-1)}{k!}\right]{\left(\frac{p}{\mu }\right)}^{i-2}$$

$$+\sum_{s=3}^{k-1}{\left(-1\right)}^{-s}\left[\prod_{j=2}^{s-1}\left(i-j\right)\right]\left[\begin{array}{c} \prod_{j=0}^{k-(s+1)}\left(i+j\right) \end{array}\right]\left[\frac{-(i-s)(i+(k-1))}{(s-1)!k(k-s)!}+\frac{\left(i-(k+1))(i+(k-s)\right)}{(s-2)!k(k-(s-1))!}-\frac{\left(i-1)(i-s\right)}{(s-2)!k(k-(s-1))!}\right]{\left(\frac{p}{\mu }\right)}^{i-s}$$

$$+(\frac{\left(i+(k-1)\right)}{{\left(-1\right)}^{k-1}k!}\left[\prod_{j=1}^{k-1}\left(i-1-j\right)\right]+\frac{(i-(k+1))\left[\prod_{j=1}^{k-2}(i-1-j)\right]i}{{\left(-1\right)}^{k-2}(k-2)!k}-\frac{(i-2)(i-1)\left[\prod_{j=1}^{k-2}\left(i-2-j\right)\right]}{{\left(-1\right)}^{k-2}(k-2)!k}){\left(\frac{p}{\mu }\right)}^{i-k}$$

$$=\left[\prod_{j=0}^{k-1}\left(i+j\right)\right]\frac{{\left(\frac{p}{\mu }\right)}^{i-1}}{k!}+\left[\prod_{j=0}^{k-3}\left(i+j\right)\right]\left[\frac{-i^{2}-(k-3)i+(k-2)}{k(k-2)!}\right]{\left(\frac{p}{\mu }\right)}^{i-2}$$

$$+\sum_{s=3}^{k-1}{\left(-1\right)}^{-s}\left[\begin{array}{c} \prod_{i=2}^{s-1}\left(i-j\right) \end{array}\right]\left[\begin{array}{c} \prod_{j=0}^{k-(s+1)}\left(i+j\right) \end{array}\right]{\left(\frac{p}{\mu }\right)}^{i-s}\left[\frac{-(k-(s-1))(i-s)(i+(k-1))+(s-1)(i-(k+1))(i+(k-s))-(s-1)(i-1)(i-s)}{(s-1)!k(k-(s-1))!}\right]$$

$$+{\left(\frac{p}{\mu }\right)}^{i-k}{\left(-1\right)}^{-k}\left(\prod_{j=2}^{k-1}\left(i-j\right)\right)\left[\frac{-(i+(k-1))(i-k)+(k-1)(i-(k+1))i-(i-1)(i-k)(k-1)}{k!}\right]$$

$$=\left[\prod_{j=0}^{k-1}\left(i+j\right)\right]\frac{{\left(\frac{p}{\mu }\right)}^{i-1}}{k!}-\left[\prod_{j=0}^{k-3}\left(i+j\right)\right]\left[\frac{\left(i+(k-2))(i-1\right)}{k(k-2)!}\right]{\left(\frac{p}{\mu }\right)}^{i-2}+{\left(-1\right)}^{k}\left(\prod_{j=2}^{k-1}\left(i-j\right)\right)\left[\frac{-i^{2}+i}{k!}\right]{\left(\frac{p}{\mu }\right)}^{i-k}$$

$$+\sum_{s=3}^{k-1}\left[\prod_{j=2}^{s-1}\left(i-j\right)\right]\left[\begin{array}{c} \prod_{j=0}^{k-(s+1)}\left(i+j\right) \end{array}\right]{\left(-1\right)}^{-s}{\left(\frac{p}{\mu }\right)}^{i-s}\left[\frac{-(k-(s-1))i^{2}-(k-(s-1))(k-(s+1))i+(k-s)(k-s+1)}{(s-1)!k(k-(s-1))!}\right]$$

$$=\left[\prod_{j=0}^{k-1}\left(i+j\right)\right]\frac{{\left(\frac{p}{\mu }\right)}^{i-1}}{k!}-\left[\prod_{j=0}^{k-2}\left(i+j\right)\right]\frac{\left(i-1\right)}{k(k-2)!}{\left(\frac{p}{\mu }\right)}^{i-2}+{\left(-1\right)}^{-(k-1)}\left(\prod_{j=2}^{k-1}\left(i-j\right)\right)\frac{i(i-1)}{k!}{\left(\frac{p}{\mu }\right)}^{i-k}$$

$$+\sum_{s=3}^{k-1}\left[\prod_{j=2}^{s-1}\left(i-j\right)\right]\left[\begin{array}{c} \prod_{j=0}^{k-(s+1)}\left(i+j\right) \end{array}\right]\left[\frac{-i^{2}-(k-(s+1))i+(k-s)}{(s-1)!k(k-s)!}\right]\frac{{\left(\frac{p}{\mu }\right)}^{i-s}}{{\left(-1\right)}^{s}}$$

$$=\left[\prod_{j=0}^{k-1}\left(i+j\right)\right]\frac{{\left(\frac{p}{\mu }\right)}^{i-1}}{k!}-\left[\prod_{j=0}^{k-2}\left(i+j\right)\right]\frac{\left(i-1\right)}{k(k-2)!}{\left(\frac{p}{\mu }\right)}^{i-2}+{\left(-1\right)}^{-(k-1)}\left(\prod_{j=0}^{k-1}\left(i-j\right)\right)\frac{{\left(\frac{p}{\mu }\right)}^{i-k}}{k!}$$

$$+\sum_{s=3}^{k-1}\left[\prod_{j=1}^{s-1}\left(i-j\right)\right]\left[\prod_{j=0}^{k-s}\left(i+j\right)\right]\frac{{\left(\frac{p}{\mu }\right)}^{i-s}}{{\left(-1\right)}^{s-1}(s-1)!k(k-s)!}$$

$$=\left[\prod_{j=0}^{k-1}\left(i+j\right)\right]\frac{{\left(\frac{p}{\mu }\right)}^{i-1}}{k!}+\sum_{s=2}^{k-1}\left[\begin{array}{c} \prod_{j=1}^{s-1}\left(i-j\right) \end{array}\right]\left[\prod_{j=0}^{k-s}\left(i+j\right)\right]\frac{{\left(\frac{p}{\mu }\right)}^{i-s}}{{\left(-1\right)}^{s-1}(s-1)!k(k-s)!}+\left[\prod_{j=0}^{k-1}\left(i-j\right)\right]\frac{{\left(\frac{p}{\mu }\right)}^{i-k}}{{\left(-1\right)}^{k-1}k!}$$

$$=i\left[\begin{array}{c} {}[\prod_{j=1}^{k-1}\left(i+j\right) \end{array}\right]\frac{{\left(\frac{p}{\mu }\right)}^{i-1}}{k!}+\sum_{s=2}^{k-1}\left[\prod_{j=1}^{s-1}\left(i-j\right)\right]\left[\prod_{j=1}^{k-s}\left(i+j\right)\right]\frac{{\left(\frac{p}{\mu }\right)}^{i-s}}{{\left(-1\right)}^{s-1}(s-1)!k(k-s)!}+\left[\prod_{j=1}^{k-1}\left(i-j\right)\right]\frac{{\left(\frac{p}{\mu }\right)}^{i-k}}{{\left(-1\right)}^{k-1}k!}]=iy_{k}^{i}$$
